# Phosphorylated NFS1 weakens oxaliplatin-based chemosensitivity of colorectal cancer by preventing PANoptosis

**DOI:** 10.1038/s41392-022-00889-0

**Published:** 2022-02-28

**Authors:** Jin-Fei Lin, Pei-Shan Hu, Yi-Yu Wang, Yue-Tao Tan, Kai Yu, Kun Liao, Qi-Nian Wu, Ting Li, Qi Meng, Jun-Zhong Lin, Ze-Xian Liu, Heng-Ying Pu, Huai-Qiang Ju, Rui-Hua Xu, Miao-Zhen Qiu

**Affiliations:** 1 Department of Medical Oncology, Sun Yat-sen University Cancer Center, State Key Laboratory of Oncology in South China, Collaborative Innovation Center for Cancer Medicine, Sun Yat-sen University, 510060 Guangzhou, P. R. China; 2 Research Unit of Precision Diagnosis and Treatment for Gastrointestinal Cancer, Chinese Academy of Medical Sciences, 510060 Guangzhou, P. R. China; 3 Guangdong Provincial Key Laboratory of Colorectal and Pelvic Floor Disease, The Sixth Affiliated Hospital (Guangdong Gastrointestinal and Anal Hospital), Sun Yat-sen University, 510655 Guangzhou, P. R. China; 4 Department of Pathology, Sun Yat-sen University Cancer Center, 510060 Guangzhou, P. R. China; 5 Department of Gastroenterology and Urology, Hunan Cancer Hospital/The Affiliated Cancer Hospital of Xiangya School of Medicine, Central South University, 410013 Changsha, P. R. China; 6 Department of Colorectal Surgery, Sun Yat-sen University Cancer Center, 510060 Guangzhou, P. R. China

**Keywords:** Cancer metabolism, Tumour biomarkers, Gastrointestinal cancer

## Abstract

Metabolic enzymes have an indispensable role in metabolic reprogramming, and their aberrant expression or activity has been associated with chemosensitivity. Hence, targeting metabolic enzymes remains an attractive approach for treating tumors. However, the influence and regulation of cysteine desulfurase (NFS1), a rate-limiting enzyme in iron–sulfur (Fe–S) cluster biogenesis, in colorectal cancer (CRC) remain elusive. Here, using an in vivo metabolic enzyme gene-based clustered regularly interspaced short palindromic repeats (CRISPR)-Cas9 library screen, we revealed that loss of NFS1 significantly enhanced the sensitivity of CRC cells to oxaliplatin. In vitro and in vivo results showed that NFS1 deficiency synergizing with oxaliplatin triggered PANoptosis (apoptosis, necroptosis, pyroptosis, and ferroptosis) by increasing the intracellular levels of reactive oxygen species (ROS). Furthermore, oxaliplatin-based oxidative stress enhanced the phosphorylation level of serine residues of NFS1, which prevented PANoptosis in an S293 phosphorylation-dependent manner during oxaliplatin treatment. In addition, high expression of NFS1, transcriptionally regulated by MYC, was found in tumor tissues and was associated with poor survival and hyposensitivity to chemotherapy in patients with CRC. Overall, the findings of this study provided insights into the underlying mechanisms of NFS1 in oxaliplatin sensitivity and identified NFS1 inhibition as a promising strategy for improving the outcome of platinum-based chemotherapy in the treatment of CRC.

## Introduction

Chemotherapy remains one of the main nonsurgical tumor control approaches in patients with unresectable colorectal cancer (CRC).^1^ However, associated side effects and chemosensitivity are the main contributors to treatment failure.^2^ Therefore, the development of effective strategies for enhancing chemosensitivity is urgently needed to improve the survival and prognosis of patients with CRC.

Cells carry out multiple regulated cell death programs, via extensive crosstalk that can be activated simultaneously under specific conditions. This fact is consistent with the recently proposed concept of “PANoptosis”.^3^ Interferon regulatory factor 1 (IRF1), TNF-α, and IFN-γ have been shown to induce PANoptosis to prevent tumorigenesis, particularly in CRC colitis-associated tumorigenesis.^4,5^ However, the role of PANoptosis in tumor chemosensitivity has not been elucidated.

Metabolic reprogramming is a crucial hallmark of malignant cells.^6,7^ Tumors develop fixed dependencies to reprogram metabolic activities, which can be exploited for the diagnosis, monitoring, and treatment of tumors.^8,9^ An increasing number of studies indicate that several inhibitors targeting these metabolic pathways exhibit effectiveness in tumor suppression, and these strategies have thus advanced into clinical trials.^10^ Considering that metabolic enzymes have an indispensable role in metabolic reprogramming and their aberrant expression or activity are closely related to tumorigenesis, tumor progression, and chemosensitivity,^10,11^ therefore, targeting and inhibiting these metabolic enzymes could significantly increase the chemosensitivity of tumor cells.

Iron–sulfur (Fe–S) clusters are essential cofactors of Fe–S proteins, which are involved in a multitude of cellular processes, including iron homeostasis, energy metabolism, and lipid biosynthesis.^12,13^ Defects in Fe–S cluster biogenesis can cause metabolic diseases and even affect the tumorigenesis and development of tumors in humans.^14,15^ During periods of rapid cancer cell growth, increased Fe–S cluster turnover increases Fe–S cluster biosynthesis to replenish Fe–S cluster storage, especially under external stimuli, such as redox stress.^16^ However, the role and mechanism of Fe–S cluster metabolism in tumor chemosensitivity is not fully understood, and further research is needed.

In this study, using an in vivo metabolic enzyme gene-based CRISPR-Cas9 screen, we revealed that cysteine desulfurase (NFS1) deficiency synergizing with oxaliplatin treatment triggered PANoptosis by increasing intracellular oxidative stress. NFS1 prevented PANoptosis in an S293 phosphorylation-dependent manner under oxaliplatin treatment. We also demonstrated that NFS1 was transcriptionally regulated by MYC and that high NFS1 expression in patients with CRC was associated with a poor prognosis. Overall, our study revealed NFS1 inhibition as an actionable strategy for improving the antitumor efficacy of platinum-based chemotherapy in CRC treatment.

## Results

### In vivo CRISPR screening reveals that NFS1 deficiency enhances the sensitivity of CRC cells to oxaliplatin

**Fig. 1:**
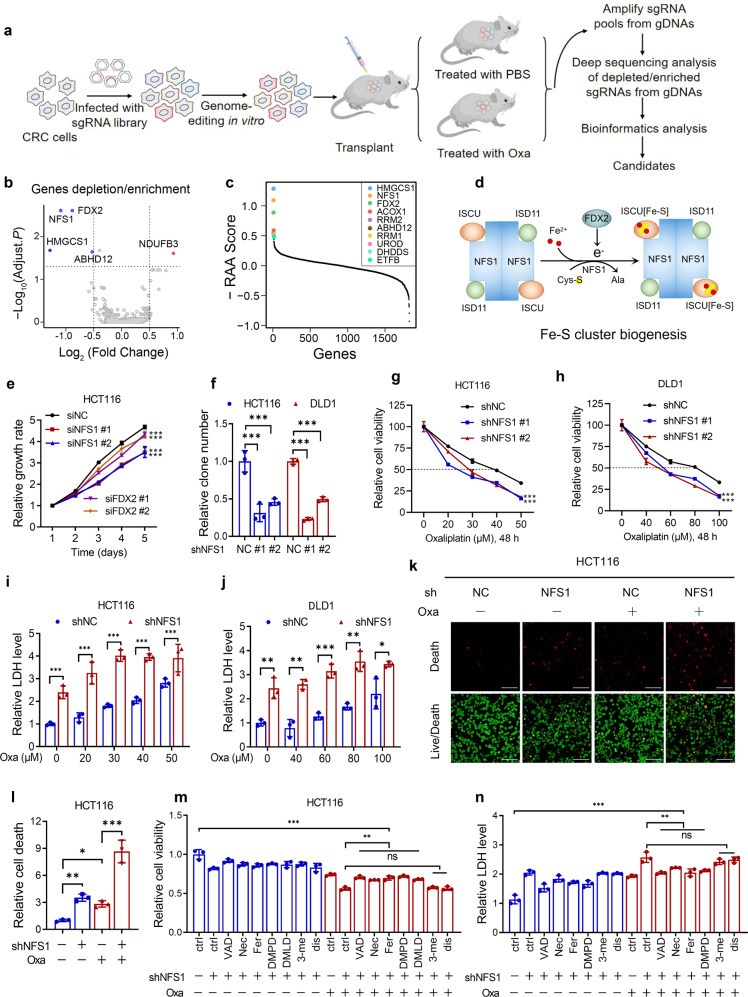
In vivo CRISPR screening reveals that NFS1 deficiency enhances the sensitivity of CRC cells to oxaliplatin (Oxa). **a** Diagram showing the strategy of the CRISPR-based screen in vivo (*n* = 6). **b** Volcano plot illustrating the depleted or enriched genes in the oxaliplatin-treatment group compared with the control group based on the depletion or enrichment of sgRNAs. Each dot represents a gene whose knockout can enhance (blue) or reduce (red) the sensitivity of cells to oxaliplatin treatment. **c** Illustration of the top ten candidates depleted in the oxaliplatin-treatment group. The analyzed CRISPR screening data are provided in Supplementary Table S5. **d** Schematic illustration of Fe–S cluster biogenesis and the main enzymes involved in this process. **e** MTS analysis of the proliferation of HCT116 cells in which *NFS1* or *FDX2* is silenced. **f** Quantification of colony formation analysis reflecting the proliferation of control and *NFS1*-knockdown HCT116 and DLD1 cells. **g**, **h** Cell viability of HCT116 and DLD1 cells treated with different concentrations of oxaliplatin for 48 h after *NFS1* knockdown. **i**, **j** LDH analysis indicating the cytotoxicity of different concentrations of oxaliplatin for 48 h in HCT116 and DLD1 cells with *NFS1* knockdown. **k** Live/dead viability/cytotoxicity assay showing the dead (red) and live (green) cells among control and *NFS1*-knockdown HCT116 cells treated with 40 µM oxaliplatin for 24 h. Scale bar = 100 μm. **l** Quantification of the relative number of dead cells in (**k**). **m**, **n** Cell viability (**m**) and cytotoxicity (**n**) assessments of control and *NFS1*-knockdown HCT116 cells treated or not treated with 40 µM oxaliplatin for 24 h in combination with the apoptosis inhibitor Z-VAD-FMK (VAD, 25 µM), the necroptosis inhibitor necrostatin (Nec, 20 µM), the ferroptosis inhibitor ferrostatin-1 (Fer, 10 µM), the pyroptosis inhibitors Ac-DMPD/DMLD-CMK (DMPD/DMLD, 20 µM) and disulfiram (dis, 1 µM) or the autophagy inhibitor 3-methyladenine (3-me, 10 µM). The data in (**e**–**j**) and (**l**–**n**) are representative of three independent experiments and presented as the mean ± SD. The *P* values in (**e**–**h**) were calculated by two-way ANOVA with Dunnett’s multiple comparisons test, those in (**l**–**n**) were calculated by one-way ANOVA with Tukey’s multiple comparisons test, and those in (**i**, **j**) were calculated by two-tailed unpaired Student’s *t* test. *P < 0.05, **P < 0.01, ***P < 0.001

To identify cell-intrinsic metabolic enzymes that synergize with chemotherapy in CRC, we performed a metabolic enzyme gene-based CRISPR-Cas9 screen of transplantable tumors treated with control or oxaliplatin, which is the backbone agent for patients with CRC in both adjuvant and metastatic settings^17,18^ (Fig. 1a and Supplementary Fig. S1a, b). Compared with the control, we showed the depleted and enriched genes in the oxaliplatin-treatment group in Fig. 1b. An inspection of the depleted genes revealed that their loss increased the sensitivity of tumor cells to oxaliplatin-based chemotherapy (Supplementary Fig. S1c). Of note, *NFS1* and *ferredoxin-2* (*FDX2/FDX1L*) were among the most depleted genes in the oxaliplatin-treatment group (Fig. 1c, Supplementary Fig. S1d, and Supplementary Table S5). As NFS1 and FDX2 are two essential metabolic enzymes in Fe–S cluster biosynthesis whereby NFS1 harvests sulfur from cysteine residues and FDX2 transfers electrons for the initial step^19^ (Fig. 1d), which suggests that NFS1 and FDX2 could induce oxaliplatin resistance in CRC.

To evaluate the function of NFS1 and FDX2 in the behaviors of CRC cells, we silenced *NFS1* and *FDX2* in HCT116 and DLD1 CRC cells (Fig. S1e) and found that NFS1 inhibition exerted a better suppressive effect on cell growth (Fig. 1e and Supplementary Fig. S1f). Knocking down *NFS1* (Supplementary Fig. S1g) successfully reduced cytoplasmic aconitase (ACO1) activity (Supplementary Fig. S1h), which is a sensitive measure of Fe–S cluster synthetic capacity,^12^ and impaired colony formation (Fig. 1f and Supplementary Fig. S1i). Correspondingly, *FDX2* silencing also inhibited colony formation and reduced the activity of ACO1 but exerted a weaker inhibitory effect than that observed with NFS1 suppression (Supplementary Fig. S1i–k). The different effects between cells could be due to the different knockdown efficiency. To verify the role of NFS1 in oxaliplatin treatment, we treated CRC cells with different concentrations of oxaliplatin and found that NFS1 knockdown exerted a significant synergistic effect with oxaliplatin to notably reduce cell viability (Fig. 1g, h) and increase cell cytotoxicity, as demonstrated through an analysis of lactate dehydrogenase (LDH) activity (Fig. 1i, j). The synergistic effects were evaluated through a Bliss test^20^ (Supplementary Fig. S1l). In addition, compared with the results observed with the control cells, the cells in which NFS1 was inhibited exhibited increased cell death, and this increase was more pronounced when the inhibition was combined with oxaliplatin treatment (Fig. 1k, l and Supplementary Fig. S1m, n). Collectively, these data support the CRISPR-Cas9 screening results and suggest that NFS1 depletion enhances the antitumor effect of oxaliplatin.

To investigate the type of cell death programs that occurred, multiple inhibitors of common cell death pathways, including apoptosis, necroptosis, ferroptosis, pyroptosis, and autophagy,^12,21,22^ were used to rescue the cell death induced by NFS1 suppression combined with oxaliplatin treatment. Notably, the apoptosis inhibitor Z-VAD-FMK, the necroptosis inhibitor necrostatin-1, the ferroptosis inhibitor ferrostatin-1, and the pyroptosis inhibitor Ac-DMPD/DMLD-CMK that inhibits gasdermin E (GSDME), reversed the reduced cell viability and enhanced cell cytotoxicity induced by NFS1 deficiency under oxaliplatin treatment, but this finding was not obtained with the autophagy inhibitor 3-methyladenine or the pyroptosis inhibitor disulfiram, which blocks GSDMD (Fig. 1m, n and Supplementary Fig. S1o, p). However, none of the inhibitors resulted in complete recovery to the levels observed in the control group (Fig. 1m, n and Supplementary Fig. S1o, p). Therefore, we hypothesized that NFS1 inhibition synergizes with oxaliplatin to simultaneously trigger multiple forms of cell death (PANoptosis).

### NFS1 deficiency synergizes with oxaliplatin treatment to induce PANoptosis

**Fig. 2:**
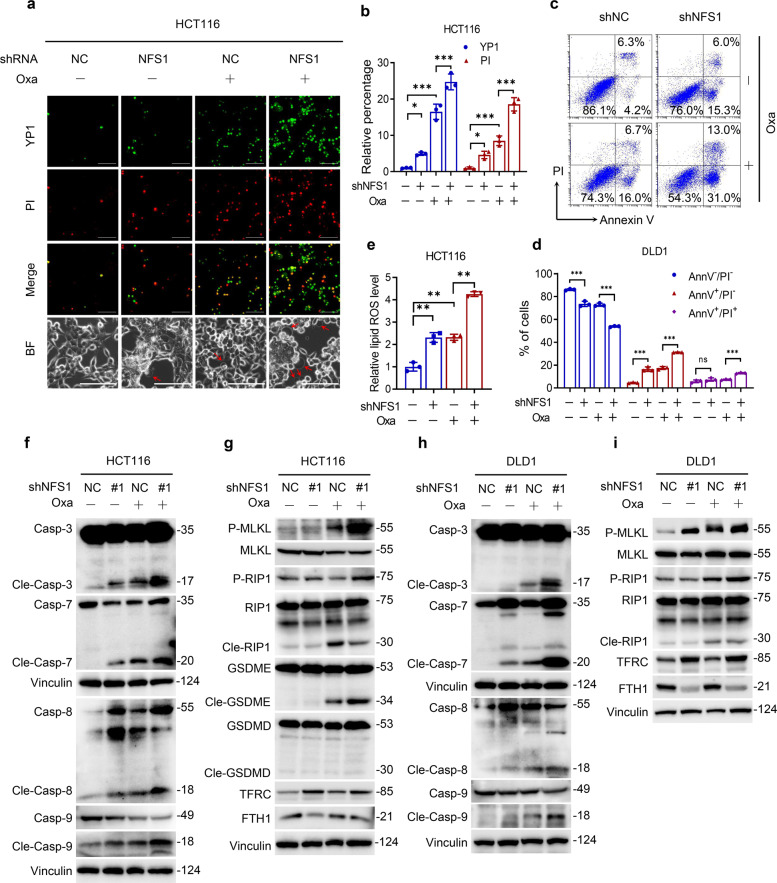
NFS1 deficiency synergizes with oxaliplatin treatment to induce PANoptosis. **a** Representative images showing YP1^+^ cells (green) that may undergo apoptosis or necroptosis and PI^+^ cells (red) that may undergo apoptosis, necroptosis, pyroptosis, or ferroptosis in control and *NFS1*-knockdown HCT116 cells treated with 40 µM oxaliplatin for 24 h. The bottom panel shows representative bright fields, and the red arrowheads indicate the large bubbles emerging from the plasma membrane. Scale bar = 100 μm. **b** Quantification of the YP1^+^ and PI^+^ cells from (**a**). **c**, **d** Flow cytometry (**c**) and quantification analysis (**d**) with Annexin V/PI staining evaluating the percentages of live cells (Annexin V^−^/PI^−^), early apoptotic cells (Annexin V^+^/PI^−^) and late apoptotic cells (Annexin V^+^/PI^+^) among the control and *NFS1*-knockdown DLD1 cells treated with PBS or oxaliplatin (80 μM, 24 h). **e** The lipid ROS levels in control and *NFS1*-knockdown HCT116 cells treated with oxaliplatin (40 μM, 24 h) were assessed by the BODIPY™ 581/591 C11 probe assay. **f**–**i** Western blotting analysis of caspase-3, cleaved caspase-3, caspase-7, cleaved caspase-7, caspase-8, cleaved caspase-8, caspase-9, cleaved caspase-9, phosphorylated MLKL, total MLKL, phosphorylated RIP1, total RIP1, cleaved RIP1, GSDME, cleaved GSDME, GSDMD, cleaved GSDMD, TFRC, FTH1 expression in control and *NFS1*-knockdown HCT116 (**f**, **g**) and DLD1 (**h**, **i**) cells treated with oxaliplatin (40 μM for HCT116 and 80 μM for DLD1, 24 h), GSDME and GSDMD are not expressed in DLD1 cells. Vinculin was included as a loading control. The data in (**b**, **d**, **e**) are representative of three independent experiments and presented as the mean ± SD. The *P* values in (**b**, **d**) were calculated by two-way ANOVA and those in (**e**) were calculated by one-way ANOVA with Tukey’s multiple comparisons test. *P < 0.05, **P < 0.01, ***P < 0.001

To further confirm the occurrence of PANoptosis, we found that *NFS1* knockdown combined with oxaliplatin treatment significantly increased the number of dead cells, including YP1-positive cells that are indicative of cellular apoptosis or necroptosis and PI-positive cells that are indicative of cellular necroptosis, pyroptosis or ferroptosis (Fig. 2a, b and Supplementary Fig. S2a, b). The analysis of HCT116 cells also shows that the number of large bubbles emerging from the plasma membrane was markedly higher in the combination group than in the negative control group, the NFS1 knockdown group, and the oxaliplatin-treatment group, which indicated the occurrence of pyroptosis (Fig. 2a). In addition, NFS1 depletion followed by oxaliplatin treatment quantitatively increased the number of early/late apoptotic cells and notably reduced the number of live cells (Fig. 2c, d and Supplementary Fig. S2c, d). In addition, a greater amount of lipid ROS was detected in the NFS1-knockdown group and the oxaliplatin-treatment group than in the control group. This increase was even more pronounced in the combination group, suggesting the occurrence of ferroptosis (Fig. 2e and Supplementary Fig. S2e, f). Hence, these data demonstrate that NFS1 deficiency in combination with oxaliplatin contributes to the activation of PANoptosis.

We further investigated how NFS1 inhibition affected these cell death pathways at the molecular level during oxaliplatin treatment. For apoptosis,^23^ we found markedly increased cleaved forms of executioner caspase-3 and caspase-7 CRC cells treated with oxaliplatin after *NFS1* knockdown (Fig. 2f, h). As upstream factors of activated caspase-3 and caspase-7, the initiators caspase-8 and caspase-9 also exhibited similar cleavage patterns (Fig. 2f, h). Cells with *NFS1* knockdown and oxaliplatin treatment showed robust phosphorylation of pseudokinase mixed lineage kinase-like domain (MLKL), an inducer of necroptosis^3^ (Fig. 2g, i). Accordingly, we noted enhanced levels of phosphorylated RIPK1 and cleaved RIPK1 (Fig. 2g, i), an upstream molecule that induces the phosphorylation of MLKL and participates in the crosstalk between apoptosis and necroptosis.^24^

Next, we explored the impact of the treatments on markers of pyroptosis. GSDMD and GSDME are the main executioners that form membrane pores under specific conditions.^25^ The results revealed no obvious effect on GSDMD, but significant activation and cleavage of GSDME were observed in the combination group and increased activation of caspase-3 and caspase-7 that can increase the activity of GSDME^26^ (Fig. 2g), which were consistent with our previous results (Fig. 1m, n). Moreover, the expression of GSDMD and GSDME was visibly detected only in HCT116 cells but not in DLD1 cells, which was in line with the increased number of large bubbles observed only in HCT116 cells (Fig. 2a and Supplementary Fig. S2a). In addition, ferroptosis is characterized by iron accumulation and lipid ROS induction.^12^ Accordingly, *NFS1* knockdown robustly increased the transferrin receptor 1 (TFRC) level and reduced the translation of ferritin heavy chain (FTH1) level which function as iron-responsive proteins to induce iron accumulation in CRC cells lacking NFS1 (Fig. 2g, i). However, no obvious difference in the TFRC and FTH1 levels was found between the combination group and the *NFS1*-knockdown group, which demonstrates that oxaliplatin treatment exerts little effect on the iron-starvation response (Fig. 2g, i).

Studies have revealed that various cell death programs play alternating roles and exhibit extensive crosstalk, which results in resistance to one pathway sensitizing cells to death via another pathway in a specific environment.^27,28^ We thereby blocked one component of PANoptosis to explore the effect on other components and found that the silencing of *caspase-3*, *caspase-7*, or *TFRC* in CRC cells robustly increased the phosphorylation of MLKL, which may be caused by the heterogeneity among cells (Supplementary Fig. S2g–j). CRC cells in which NFS1 suppression synergized with oxaliplatin treatment underwent PANoptosis, including apoptosis, necroptosis, pyroptosis, and ferroptosis. This led us to speculate NFS1 could be a widespread target for susceptibility to oxaliplatin-based chemotherapy.

### NFS1 deficiency enhances the antitumor effect of oxaliplatin in vivo

**Fig. 3:**
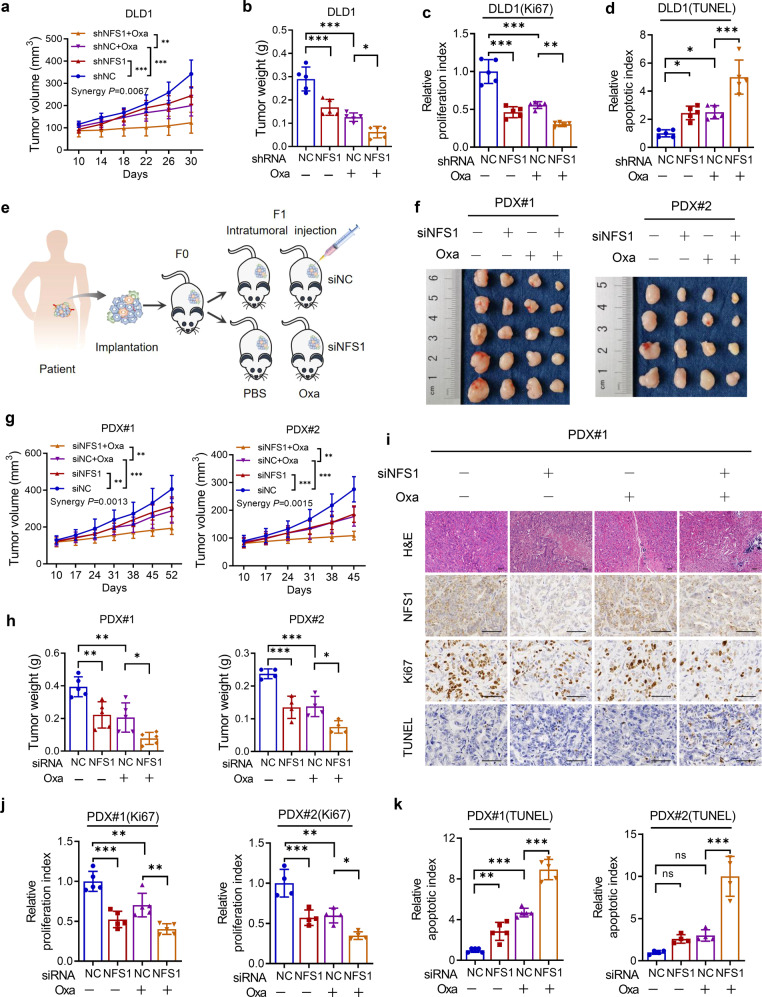
NFS1 deficiency enhances the antitumor effect of oxaliplatin in vivo. **a**, **b** Statistical analysis of CDX tumor volumes (**a**) and weights (**b**) in nude mice after implantation of *NFS1*-knockdown or control DLD1 cells, followed by i.p. injection of oxaliplatin (7.5 mg/kg) or PBS (*n* = 5, Bliss synergy *P* value is shown). **c**, **d** Quantification of the proliferation index (Ki67 staining) (**c**) and apoptotic index (TUNEL staining) (**d**) of DLD1-based xenograft tumors. **e** Illustration of the methodology used to establish CRC PDX models. **f** Photographs of the excised tumors from PDX #1 (left) and PDX #2 (right) models after intratumoral injection of in vivo-optimized NFS1 inhibitor (si*NFS1*) or the control, followed by i.p. injection of oxaliplatin (7.5 mg/kg) or PBS (PDX #1, *n* = 5; PDX #2, *n* = 4) and comparison of the tumor sizes. **g**, **h** Statistical analysis of the tumor volumes (**g**, Bliss synergy *P* values are shown) and weights (**h**) in nude mice from the PDX #1 (left) and PDX #2 (right) models. **i** Representative H&E and IHC staining images of NFS1, Ki67, and TUNEL in PDX #1-based paraffin-embedded subcutaneous tumor sections. Scale bar = 50 μm. **j**, **k** Quantification of the proliferation index (Ki67 staining) (**j**) and apoptotic index (TUNEL staining) (**k**) of the PDX #1 (left) and PDX #2 (right) models. The data in (**a**–**d**, **g**, **h**, **j**, **k**) (PDX #1) are representative of five independent experiments and those in (**g**, **h**, **j**, **k**) (PDX #2) are representative of four independent experiments. All the data are presented as mean ± SD. The *P* values in (**a**, **g**) were calculated by two-way ANOVA, and those in (**b**–**d**, **h**, **j**, **k**) were calculated by one-way ANOVA with Tukey’s multiple comparisons test. *P < 0.05, **P < 0.01, ***P < 0.001

To investigate the role of NFS1 deficiency in tumorigenesis and chemotherapy efficiency in vivo, we subcutaneously implanted normal or NFS1-deficient cells into mice and then treated the mice with PBS or oxaliplatin. As expected, the combination of NFS1 depletion and oxaliplatin treatment significantly reduced tumor growth and the xenograft tumor weight and achieved the best suppressive effects against tumorigenic activities. The synergy *P* value was calculated to represent the synergistic effect^29^ (Supplementary Fig. S3a and Fig. 3a, b). In addition, *NFS1* knockdown reduced cell proliferation (Ki67 staining), and enhanced cell death (TUNEL staining), and these effects were more pronounced in the combination group (Supplementary Fig. S3b and Fig. 3c, d). These results are consistent with the in vitro results.

To better emulate the physical tumor microenvironment, we established two patient-derived xenograft (PDX) tumor models (Fig. 3e and Supplementary Fig. S3c, d). Consistently, we observed attenuated tumor weights and volumes after silencing *NFS1* compared with those in the control group, whereas NFS1 deficiency combined with oxaliplatin treatment also exerted a synergistic effect (Fig. 3f–h). Synergy *P* values were calculated to represent the synergistic effects^29^ (Fig. 3a, g). The pathological analysis revealed increased necrosis and fibrosis in the NFS1-depleted tumors (Fig. 3i and Supplementary Fig. S3e). The combination treatment group exhibited the highest levels of necrosis and fibrosis, and even exhibited calcification and foam cells, which indicated a strong therapeutic response^30^ (Fig. 3i and Supplementary Fig. S3e). Similarly, the combination treatment group showed decreased cell proliferation and increased cell death (Fig. 3j, k). Taken together, the above-described results further highlight the synergistic effect of NFS1 deficiency with oxaliplatin treatment in vivo.

### Oxidative stress is critical for NFS1 deficiency-induced PANoptosis under oxaliplatin treatment

**Fig. 4:**
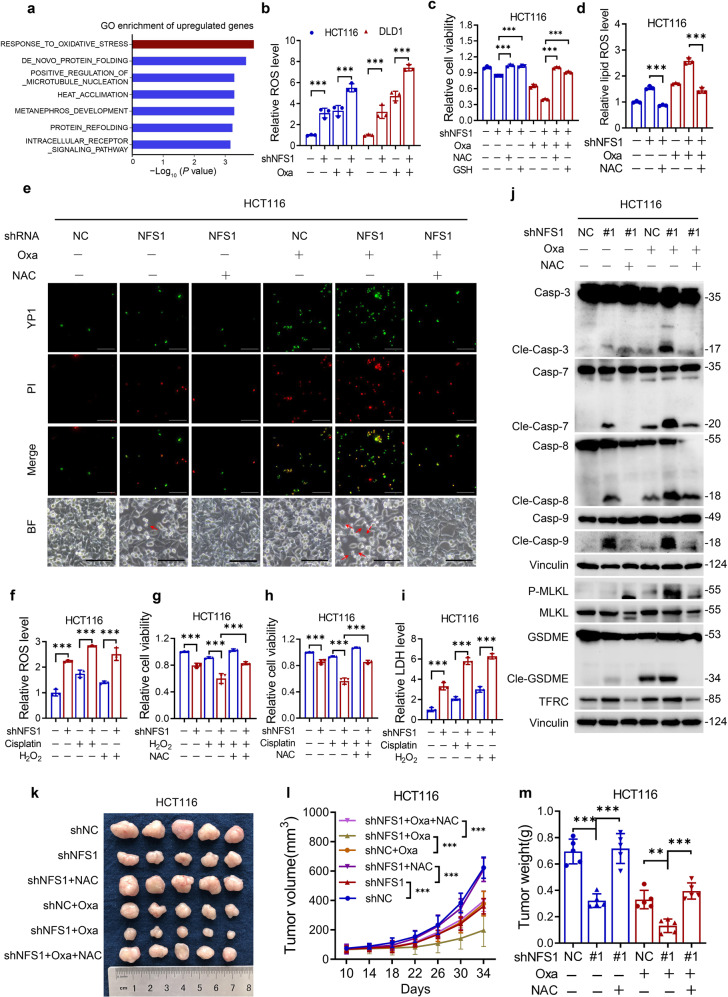
Oxidative stress is critical for NFS1 deficiency-induced PANoptosis under oxaliplatin treatment. **a** Gene Ontology (GO) enrichment analysis of genes that exhibited twofold upregulation under oxaliplatin treatment (40 μM, 24 h) in the NFS1-knockdown group compared with the control group. **b** ROS analysis of control and *NFS1*-knockdown HCT116 and DLD1 cells under PBS or oxaliplatin treatment (40 μM/80 μM, 24 h). **c** Cell viability of HCT116 cells treated with 40 μM oxaliplatin combined with 5 mM NAC or 5 mM GSH for 24 h after *NFS1* knockdown. **d** Lipid ROS analysis of HCT116 cells treated with 40 μM oxaliplatin combined with 5 mM NAC for 24 h after *NFS1* knockdown. **e** Representative images showing YP1^+^ cells (green) which may undergo apoptosis or necroptosis and PI^+^ cells (red) which may undergo apoptosis, necroptosis, pyroptosis, or ferroptosis among control and *NFS1*-knockdown HCT116 cells treated with 40 μM oxaliplatin combined with 5 mM NAC (24 h) after *NFS1* knockdown. The bottom panel shows representative bright fields and the red arrowheads indicate the large bubbles emerging from the plasma membrane. Scale bar = 100 μm. **f** ROS analysis of control and *NFS1*-knockdown HCT116 cells under cisplatin (40 μM, 24 h) or H_2_O_2_ (100 μM, 24 h) treatment. **g**, **h** Cell viability of HCT116 cells treated with 100 μM H_2_O_2_ (**g**) and 40 μM cisplatin (**h**) combined with 5 mM NAC for 24 h after *NFS1* knockdown. **i** Cell cytotoxicity assessments of control and *NFS1*-knockdown HCT116 cells under cisplatin (40 μM, 24 h) or H_2_O_2_ (100 μM, 24 h) treatment. **j** Western blotting analysis of caspase-3, cleaved caspase-3, caspase-7, cleaved caspase-7, caspase-8, cleaved caspase-8, caspase-9, cleaved caspase-9, phosphorylated MLKL, total MLKL, GSDME, cleaved GSDME and TFRC expression in control and *NFS1*-knockdown HCT116 cells after treatment with 40 μM oxaliplatin combined with 5 mM NAC (24 h). **k**–**m** Photograph showing the gross comparison (**k**), tumor volumes (**l**), and weights (**m**) of control and *NFS1*-knockdown HCT116 CDX tumors in nude mice subjected to i.p. injection of oxaliplatin (7.5 mg/kg) and NAC (1 mg/ml) in the drinking water (*n* = 5). Vinculin was included as a loading control. The data in (**b**–**d**, **f**–**i**) are representative of three independent experiments and those in (**l**, **m**) are representative of five independent experiments, and all are presented as the mean ± SD. The *P* values in (**b**, **l**) were calculated by two-way ANOVA, and those in (**c**, **d**, **f**–**i**, **m**) were calculated by one-way ANOVA with Tukey’s multiple comparisons test. **P < 0.01, ***P < 0.001

We subsequently assessed the specific mechanism by which NFS1 deficiency induced PANoptosis under oxaliplatin treatment. RNA sequencing was performed to explore the signaling pathways affected by NFS1. Under oxaliplatin treatment, *NFS1* knockdown resulted in 188 twofold upregulated genes and 285 twofold downregulated genes compared with the control group (Supplementary Fig. S4a). Notably, Gene Ontology enrichment analysis of the twofold upregulated genes showed that the “response to oxidative stress” pathway was the most prominent gene set (Fig. 4a and Supplementary Fig. S4b). Most of the enriched genes were upregulated after NFS1 suppression (Supplementary Fig. S4c). Because the product of NFS1, the Fe–S cluster, is a cofactor for many proteins (including those that maintain redox homeostasis),^12^ we detected the ROS level and demonstrated that NFS1 deficiency either alone or in combination with oxaliplatin treatment markedly increased the level of ROS compared with that in the control group (Fig. 4b). To determine whether the combined treatment induced PANoptosis via ROS production, we utilized the antioxidants N-acetyl-l-cysteine (NAC) and glutathione (GSH) to neutralize the increased ROS (Supplementary Fig. S4d). Notably, the diminished cell viability obtained with NFS1 suppression with or without oxaliplatin treatment could be rescued by NAC or GSH (Fig. 4c and Supplementary Fig. S4e). In addition, the increases in the lipid ROS levels and numbers of cells undergoing apoptosis, necroptosis, and pyroptosis could also be mitigated (Fig. 4d, e and Supplementary Fig. S4f). Furthermore, NFS1 deficiency combined with the ROS inducer hydrogen peroxide (H_2_O_2_) or cisplatin also reduced cell viability and increased cell cytotoxicity via enhanced ROS (Fig. 4f–i). In addition, NAC treatment impaired the activation of caspase-3, caspase-7, caspase-8, caspase-9, phosphorylated MLKL, GSDME, and TFRC induced by NFS1 suppression in the presence or absence of oxaliplatin treatment (Fig. 4j and Supplementary Fig. S4g). In parallel, the attenuated tumor cell growth and weights could be rescued in vivo by the addition of NAC, consistent with the in vitro effects (Fig. 4k–m). Altogether, these data indicate that NFS1 deficiency in combination with platinum-based chemotherapy synergistically induces ROS, which subsequently causes PANoptosis.

### NFS1 prevents PANoptosis under oxaliplatin treatment depending on its phosphorylation level at S293

**Fig. 5:**
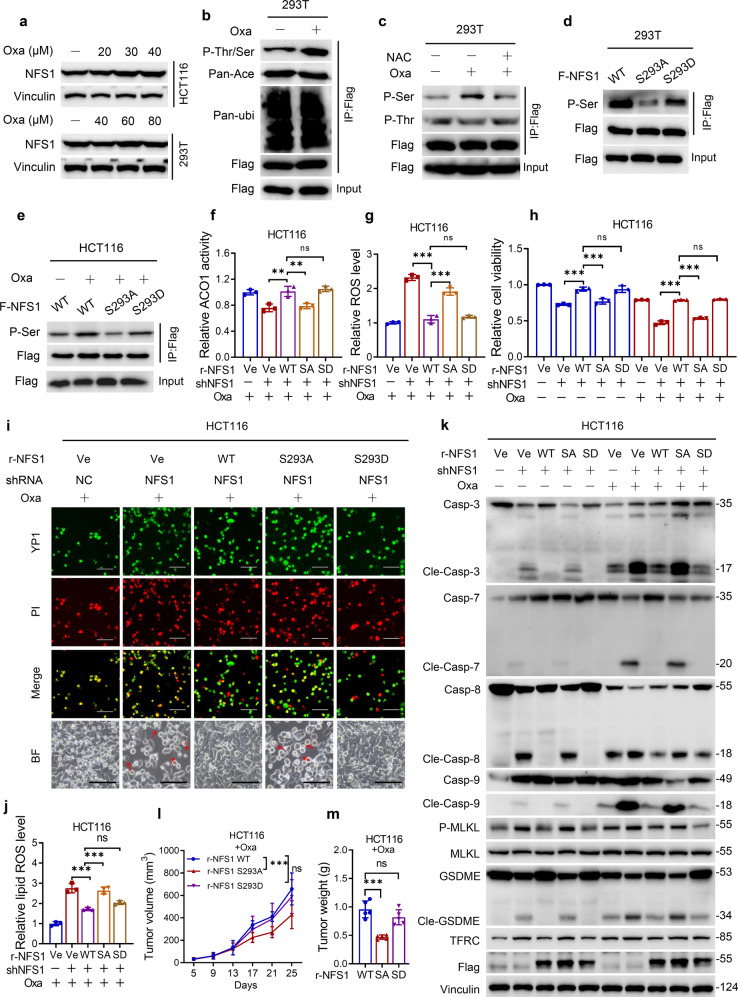
NFS1 prevents PANoptosis under oxaliplatin treatment depending on its phosphorylation level at S293. **a** Western blotting detection of NFS1 expression in HCT116 and 293T cells treated with different concentrations of oxaliplatin for 48 h. **b** IP analysis demonstrating the post-translational modifications level of NFS1 in 293T cells treated with oxaliplatin (40 μM, 24 h), including phosphorylation (P-Thr/Ser), acetylation (Pan-Ace), and ubiquitination (Pan-ubi). **c** IP analysis showing the level of phosphorylated threonine (P-Thr) and serine (P-Ser) residues of NFS1 in 293T cells treated with oxaliplatin (40 μM, 24 h). **d**, **e** IP analysis demonstrating the serine phosphorylation level of NFS1 in cells overexpressing WT *NFS1* or the S293A or S293D mutant of *NFS1* in the absence (**d**) or presence of oxaliplatin (40 μM, 24 h) (**e**). **f** ACO1 activity analysis of NFS1 in control and *NFS1*-knockdown HCT116 cells when overexpressed *rNFS1* WT and S293A or S293D mutant with oxaliplatin treatment (40 μM, 24 h). **g**–**j** ROS level (**g**), cell viability (**h**), YP1^+^ and PI^+^ cells (**i**), and lipid ROS levels (**j**) in HCT116 cells overexpressing *rNFS1* WT and S293A or S293D mutant with or without oxaliplatin treatment (40 μM, 24 h). The bottom panel in (**i**) shows representative bright fields, and the red arrowheads indicate the large bubbles emerging from the plasma membrane, Scale bar = 100 μm. **k** Western blotting analysis of caspase-3, cleaved caspase-3, caspase-7, cleaved caspase-7, caspase-8, cleaved caspase-8, caspase-9, cleaved caspase-9, phosphorylated MLKL, total MLKL, GSDME, cleaved GSDME, and TFRC expression in HCT116 cells overexpressing *rNFS1* WT or the S293A or S293D mutant in the absence or presence of oxaliplatin (40 μM, 24 h). **l**, **m** Statistical analysis of the tumor volumes (**l**) and weights (**m**) in nude mice after implantation of *NFS1*-knockdown HCT116 cells overexpressing *rNFS1* WT or the S293A or S293D mutant, followed by an i.p. injection of oxaliplatin (7.5 mg/kg) (*n* = 5). Vinculin or flag was included as a loading control. The data in (**f**–**h**, **j**) are representative of three independent experiments, and those in (**l**, **m**) are representative of five independent experiments, and all are presented as the mean ± SD. The *P* values in (**f**–**h**, **j**, **m**) were calculated by one-way ANOVA, and those in (**l**) were calculated by two-way ANOVA with Tukey’s multiple comparisons test. **P < 0.01, ***P < 0.001

Because cancer cells can modify a set of defense mechanisms, including the assembly of Fe–S clusters, to survive during redox stress^16^ and that platinum-based chemotherapy treatment could increase ROS level (Fig. 4b, f), we subsequently explored the regulatory effect of oxaliplatin on NFS1 and found that the mRNA and protein levels of NFS1 were unaffected by oxaliplatin treatment based on the sensitivity to oxaliplatin (Supplementary Fig. S5a, b and Fig. 5a). Hence, we detected common post-translational modifications of NFS1, including phosphorylation, acetylation, and ubiquitination. Oxaliplatin treatment significantly increased the phosphorylation (threonine/serine) level of NFS1 with no change in the acetylation and ubiquitination levels (Fig. 5b). Furthermore, the treatment markedly enhanced the serine but not the threonine phosphorylation level of NFS1, and this effect could be reversed by NAC (Fig. 5c). We speculated that the serine phosphorylation of NFS1 provides a rapid adaptation for Fe–S cluster demand under oxaliplatin-mediated oxidative stress. A previous study showed that the phosphorylation levels at T195, S334, and T336, which are conserved in humans, could affect the cysteine desulfurase activity of NFS1.^31^ To verify whether the level of phosphorylated S293 in human NFS1 (analogous to S334 in yeast) plays a crucial role under oxaliplatin treatment, we mutated the serine phosphorylation residue to alanine (S293A) or aspartate (S293D) (Fig. 5d). The overexpression of *NFS1* S293A blocked the oxaliplatin-enhanced phosphorylation level of NFS1 (Fig. 5e), suggesting that the phosphorylation level of NFS1 at S293 may play a crucial role in its cysteine desulfurase activity.

We re-expressed shRNA-resistant wild-type (WT), S293A, and S293D *rNFS1* in *NFS1*-knockdown cells under oxaliplatin treatment. The overexpression of *rNFS1* WT significantly reverted the reduction in ACO1 activity caused by NFS1 inhibition, and the rescued effect in cells overexpressing *rNFS1* S293A but not *rNFS1* S293D was weaker than that in *rNFS1* WT-overexpressing cells (Fig. 5f). Furthermore, overexpression of the *rNFS1* S293D mutant but not *rNFS1* S293A maintained the reduced ROS levels, increased cell viability, disturbed the cell cytotoxicity and lipid ROS levels, and reduced multiple cell death pathways, which was similar to the results obtained with *rNFS1* WT (Fig. 5g–j and Supplementary Fig. S5c, d). Consistently, the activation of caspase-3, caspase-7, caspase-8, caspase-9, phosphorylated MLKL, GSDME, and TRFC induced by NFS1 suppression with or without oxaliplatin treatment was sufficiently abrogated by the re-expression of *rNFS1* WT and S293D mutant but not S293A mutant, and this effect was more obvious under oxaliplatin treatment (Fig. 5k). In parallel, under oxaliplatin treatment, *rNFS1* S293A overexpression but not *rNFS1* S293D attenuated tumor cell growth and weights in vivo compared with the results obtained with *rNFS1* WT overexpression (Supplementary Fig. S5e and Fig. 5l, m). Taken together, the data showed that NFS1 prevents the activation of PANoptosis under oxaliplatin treatment in an S293 phosphorylation-dependent manner.

### *NFS1* is transcriptionally regulated by MYC

**Fig. 6:**
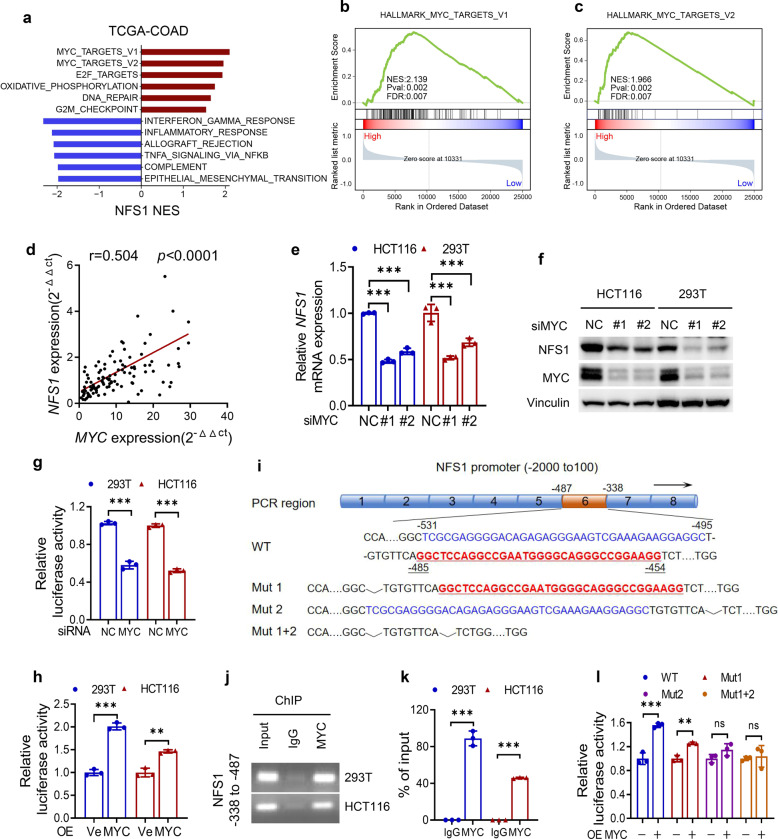
*NFS1* is transcriptionally regulated by MYC. **a** Hallmark enriched pathway analysis showing pathways associated with the expression of NFS1. **b**, **c** Gene set enrichment analysis suggesting that NFS1 is positively correlated with MYC pathways. **d** Q-PCR analysis showing that *NFS1* expression is positively correlated with *MYC* expression in CRC tumor tissues from SYSUCC (*n* = 115, Pearson’s correlation analysis). **e**, **f** Q-PCR analysis (**e**) and western blotting analysis (**f**) of NFS1 and MYC expression in HCT116 and 293T cells with control or silenced expression of MYC. **g**, **h** Dual-luciferase promoter activity analysis showing the transcriptional activity of *NFS1* in HCT116 and 293T cells with MYC downregulation (**g**) or upregulation (**h**). **i** Schematic illustration of the *NFS1* promoter containing two main MYC-binding sites (−531 to −495 and −485 to −454). The strategy for mutating the promoter is also shown. **j**, **k** Agarose gel electrophoresis (**j**) and Q-PCR (**k**) assay after ChIP analysis showing the occupancy of MYC on the *NFS1* promoter (region −478 to −338) in HCT116 and 293T cells. **l** Luciferase promoter activity analysis of *NFS1* transcriptional activity in 293T cells overexpressing *NFS1* WT or truncation mutant (**i**). Vinculin was included as a loading control. The data in (**e**, **g**, **h**, **k**, **l**) are representative of three independent experiments and presented as the mean ± SD. The *P* values in (**e**) were calculated by two-way ANOVA with Dunnett’s multiple comparisons test, those in (**g**, **h**, **k**, **l**) were calculated by two-tailed unpaired Student’s *t* test, and this in (**d**) was calculated by Pearson’s correlation analysis and chi-square test. **P < 0.01, ***P < 0.001

The transcriptional regulatory mechanism of *NFS1* in CRC remains unclear. A hallmark enriched pathway analysis suggested that the expression of *NFS1* was positively correlated with the MYC pathway (Fig. 6a–c), and *NFS1* expression was positively correlated with *MYC* expression in our CRC tumor tissues and in tissues from the TCGA database (Fig. 6d and Supplementary Fig. S5f). To verify whether MYC indeed transcriptionally regulates *NFS1* expression, we found that MYC depletion resulted in a significant decrease in the expression of NFS1 (Fig. 6e, f and Supplementary Fig. S5g), whereas the overexpression of MYC increased the expression of NFS1 at the protein and mRNA levels (Supplementary Fig. S5h, i). Moreover, dual-luciferase promoter activity analysis indicated that MYC downregulation or upregulation reduced or enhanced the transcriptional activity of NFS1, respectively (Fig. 6g, h). We subsequently revealed the specific binding regions of MYC on the promoter of *NFS1*, and found that MYC bound to the −487 to −338 region of the *NFS1* promoter (Fig. 6i–k). Combined with the prediction results,^32^ two main MYC-binding sequences were found in this region, and these sequences were deleted either individually or in combination, as shown in Fig. 6i. The overexpression of MYC increased the transcriptional activity of WT and Mut1 but not Mut2 and Mut (1 + 2) (Fig. 6l), which suggested that MYC directly regulates the transcription of NFS1 and that the specific binding regions on the NFS1 promoter may be located in Mut1 (−485 to −454).

### NFS1 is highly expressed in CRC and indicates a poor prognosis

**Fig. 7:**
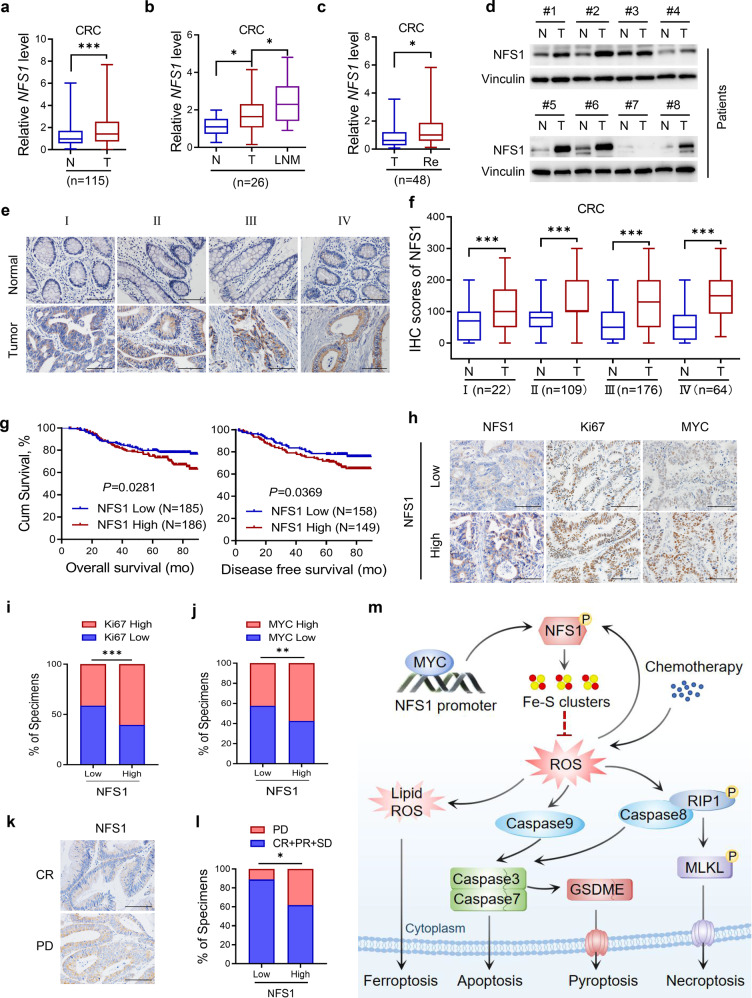
NFS1 is highly expressed in CRC and indicates poor prognosis. **a**–**c** Q-PCR detection of *NFS1* mRNA expression in 115 pairs of primary CRC tumor tissues (T) and adjacent normal tissues (N) (**a**); 26 pairs of lymph-node metastases (LNM) (**b**); and 48 pairs of CRC tissues with recurrence (Re) and without recurrence (T) (**c**)**. d** Western blotting analysis of NFS1 protein expression in eight pairs of CRC tumor tissues (T) and adjacent normal tissues (N). **e**, **f** Representative IHC staining images of NFS1. Scale bar = 100 μm (**e**); and IHC staining scores of NFS1 expression in paired primary CRC tumor tissues (T) and adjacent normal tissues (N) from patients at different cancer phases (phase I, *n* = 22; phase II, *n* = 109; phase III, *n* = 176; phase IV, *n* = 64) (**f**). All samples were obtained from SYSUCC. **g** Overall survival (left) and disease-free survival (right) assays of patients with CRC based on NFS1 protein level from (**e**, **f**)**. h** Representative IHC staining images showing NFS1, Ki67, and MYC expression in CRC tumor tissues. Scale bar = 100 μm. **i**, **j** Correlations between NFS1 expression and Ki67 (**i**) and MYC (**j**) expression (*n* = 340). **k** Representative images showing NFS1 expression in patients with FOLFOX or XELOX chemotherapy. **l** Correlation between NFS1 expression with the response of patients with CRC to FOLFOX or XELOX chemotherapy (*n* = 61, PD progressive disease, SD stable disease, PR partial response, CR complete response). **m** Proposed working model based on this study. The model shows that phosphorylated NFS1 weakens platinum-based chemosensitivity by reducing the level of ROS to prevent PANoptosis. Vinculin was included as a loading control. The data in (**a**–**c**, **f**) are presented in the form of box and whisker plots (minimum–maximum) with the horizontal line in each box representing the median, and those in (**i**, **j**, **l**) are presented as the percentage of total samples. The *P* values in (**a**, **c**, **f**) were calculated by two-tailed paired Student’s *t* test, this in (**b**) was calculated by one-way ANOVA with Tukey’s multiple comparisons test, this in (**g**) was calculated by Kaplan–Meier analysis with the log-rank test, two-sided, and those in (**i**, **j**, **l**) were calculated by Pearson’s correlation analysis and chi-square test. *P < 0.05, **P < 0.01, ***P < 0.001

We then investigated the clinical implication of NFS1 in patients with CRC. Data from the TCGA database indicated that *NFS1* is overexpressed in multiple human tumors including colon adenocarcinoma (COAD) (Supplementary Fig. S6a), and finding from our hospital cohort showed that *NFS1* mRNA was more highly expressed in CRC and esophageal squamous cell carcinoma (ESCC) tumor tissues than in matched normal tissues (Fig. 7a and Supplementary Fig. S6b). *NFS1* expression was also elevated in lymph-node metastases (Fig. 7b) and recurrent CRC tissues (Fig. 7c) in comparison with paired primary and nonrecurrent tumor tissues, respectively. Consistently, *NFS1* expression was commonly upregulated in different types of CRC tissues, as supported by the Oncomine database (Supplementary Fig. S6c). At the protein level, NFS1 was notably increased in representative CRC tissues (Fig. 7d), in primary ESCC and CRC tissues compared with adjacent normal tissues, and even at different CRC phases based on tumor tissue microarrays (Fig. 7e, f and Supplementary Fig. S6d, e) (clinicopathological information is provided in Supplementary Table S1). Moreover, high NFS1 expression levels were associated with shorter overall survival and disease-free survival in patients with CRC (Fig. 7g), even after adjusting for other prognostic factors (Supplementary Table S2). In addition, patients with higher NFS1 expression exhibited stronger expressions of Ki67 and MYC, which indicated obvious positive correlations (Fig. 7h–j). These data suggest NFS1 has a widespread prognostic indicator potential.

To validate the clinical correlation between NFS1 expression and the response of patients with CRC to oxaliplatin-based chemotherapy (FOLFOX or XELOX regimens), we found that NFS1 staining was increased in patients who exhibit a poorer benefit from standard chemotherapy (Fig. 7k). The results also showed that 1 1.1% of patients with low NFS1 expression and 38.2% of patients with high NFS1 expression were resistant to chemotherapy (PD, progressive disease) (Fig. 7l). In addition, many antioxidant enzymes, such as glutathione synthetase (GSS), γ-glutamylcysteine synthetase (GCLC/GCLM), glutathione peroxidase (GPX), thioredoxin reductase (TXNRD), and catalase (CAT), are overexpressed in certain resistant cancer cells and in patients with cancer.^33^ TCGA database showed a positive correlation between *NFS1* expression and *GSS, GCLC, GCLM, GPX2, TXNRD3*, and *CAT* expression (Supplementary Fig. S6f). These results revealed that NFS1 expression might be a reliable indicator for predicting the sensitivity of patients with CRC to oxaliplatin-based chemotherapy.

## Discussion

NFS1 plays a pivotal role in Fe–S cluster assembly and several Fe–S cluster-dependent pathways to impact tumor development.^12,34^ The depletion of NFS1 combined with the inhibition of cysteine transport results in insufficient Fe–S cluster maintenance, activation of the iron-starvation response and subsequent triggering of ferroptosis.^12^ NFS1 inhibition induces persistent DNA damage and leads to cell death in breast cancer.^34^ However, the influence and regulation of NFS1 in CRC remain elusive. In this study, we first uncovered a significant oncogenic role of NFS1, which is transcriptionally regulated by MYC, in CRC progression. High NFS1 expression was revealed in multiple tumor tissues and indicated poor survival and poor response to chemotherapy in patients with CRC, which suggested that NFS1 may be a widespread potential prognostic indicator for patients with tumor. However, the significance and regulatory mechanism in other tumors need to be further studied.

Excessive ROS production, denoted as “oxidative distress”, can induce cytotoxicity and trigger multiple forms of cell death.^35,36^ For example, the suppression of methylene tetrahydrofolate dehydrogenase 2 robustly increases ROS production and induces apoptosis.^37^ ROS-inducing drugs combined with iron boost the cellular level of ROS, which subsequently triggers the pyroptosis of melanoma cells.^38^ Our results first demonstrated that NFS1 deficiency could trigger PANoptosis and showed a synergistic effect with oxaliplatin treatment by increasing the level of ROS (Fig. 7m), but the specific molecular mechanism through which Fe–S clusters regulate the ROS level needs to be further refined.

Studies have shown that inflammatory mediators IRF1, TNF, and IFN-γ can prevent tumorigenesis by inducing PANoptosis via activation of the PANoptosome.^3–5^ The activation of the ZBP1-NLRP3 inflammasome could also induce PANoptosis and suppress cancer.^39^ However, the production of inflammasome mediated by deficiency of the innate immune sensor AIM2 has protective functions in CRC by expanding a population of tumor-initiating stem cells.^40^ Hence, the role of the inflammasome in the PANoptosome and tumorigenesis needs to be further investigated based on the specific tumor microenvironment, and the synergistic activity of inflammatory mediators, such as IRF1, TNF, and IFN-γ, with NFS1 deficiency and their significance will be a promising investigation for tumor treatment.

Some cancer cells can survive via a set of defense mechanisms, such as the upregulation of antioxidant enzymes and the modification of sulfur-based metabolism, during chemotherapy treatment, and these mechanisms enable the cells to become hyposensitive and even resistant to many chemotherapeutic drugs.^10,35,41^ Many inhibitors targeting antioxidant enzymes, such as GSS, GCLC, GCLM, GPX, TXNRD, and CAT, are undergoing clinical trials for the treatment of certain drug-resistant cancers.^33^ We also found that *NFS1* expression was positively correlated with these antioxidant enzymes. Collectively, NFS1 inhibition is a promising strategy for improving the antitumor efficacy of oxaliplatin-based chemotherapy in CRC treatment.

The post-translational modifications of metabolic enzymes play a crucial role in metabolic reprogramming, and these modifications could be exploited for the development of new therapeutic approaches for cancer treatment.^7^ Reversible protein phosphorylation is one of the most common and important post-translational modifications that affect protein stability, enzyme activity, and cellular localization in response to stimuli.^42–44^ With respect to Fe–S cluster assembly, a previous study demonstrated that phosphorylation of the scaffold protein ISCU at S14 leads to nutrient-dependent stabilization and increased Fe–S cluster biosynthesis in mammalian cells.^45^ The phosphorylation of NFS1 at T195, S334, and T336 in yeast has also been illustrated to enhance cysteine desulfurase activity.^31^ Our findings show that oxaliplatin-mediated oxidative stress enhances the serine phosphorylation level of NFS1 and that NFS1 prevents the activation of PANoptosis under oxaliplatin treatment in a S293 phosphorylation-dependent manner, which suggests a novel regulatory mechanism of NFS1 activity in humans. The study of the post-translational modifications of metabolic enzymes is significant for extending chemotherapy and immunotherapy for cancer treatment. Nevertheless, the potential mechanism through which oxaliplatin-mediated oxidative stress mediates the phosphorylation status of NFS1 and the roles of NFS1 under other chemotherapy drugs need to be further illustrated.

In conclusion, our study indicates that NFS1 is a promising prognostic indicator and that the inhibition of NFS1 through the development of targeted inhibitors may be a potential therapeutic strategy for improving the antitumor efficacy of platinum-based chemotherapy in CRC treatment.

## Materials and methods

### Ethics approval and consent to participate

After obtaining written informed consent from the participants who provided samples, our study was approved by the Medical Ethics Committee of SYSUCC and complied with the Helsinki Declaration. All animal experiments were performed based on the protocol approved by our institutional Animal Care and Use Committee of Sun Yat-sen University.

### Human tissue specimens

All human tissue specimens, including 115 pairs of normal versus CRC tissue specimens, 26 pairs of primary CRC tissues versus lymph-node metastasis tissues, 48 pairs of nonrecurrence versus recurrence CRC tissues and 35 pairs of normal versus ESCC tissue specimens for Q-PCR analysis, 371 pairs of CRC tissue specimens (100 matched lymph-node metastasis tissues), 61 CRC tissues with FOLFOX or XELOX chemotherapy for IHC staining analysis and 54 pairs of ESCC tissue specimens were obtained from the Sun Yat-sen University Cancer Center (SYSUCC, Guangzhou, China). The clinicopathological characteristics of the patients with CRC whose samples were included in the study are summarized in Supplementary Tables S1 and S2.

### RNA interference, lentivirus, and plasmid transfection

Small interfering RNAs (siRNAs) targeting NFS1, FDX2, MYC, caspase-3, caspase-7, MLKL, GSDME, and TFRC were synthesized by RiboBio (Guangzhou, China) and transfected using Lipofectamine RNAiMax (13778150, Thermo Fisher Scientific, Carlsbad, CA, USA). Lentiviruses packaging NFS1 short hairpin RNAs (shRNAs) were synthesized by OBiO Technology (Shanghai, China). CRC cells were infected by lentiviruses with polybrene and selected with puromycin (HY-B1743A, MedChemExpress) for 7 days. All the targeted sequences of siRNAs and shRNAs are summarized in Supplementary Table S3. For the plasmids, N-terminal Flag-tagged expression vectors for shRNA#1-resistant NFS1 WT, site-directed mutants S293A and S293D of NFS1, N-terminal Flag-tagged MYC, and luciferase reporter plasmids containing NFS1 WT or deletion mutants (Mut1, Mut2, Mut1 + 2) were provided by Saisofi Biotechnology Co., Ltd. (Suzhou, China). The plasmids were transfected into the cells using ViaFect Transfection Reagent (E4982, Promega, Madison, WI, USA) according to the recommended protocol.

### CRISPR-Cas9 knockout screen in vivo

The human metabolic enzyme sgRNA library targets 1,773 metabolic enzyme genes (4 sgRNAs per gene). The sgRNAs were designed and synthesized by GENEWIZ (Suzhou, China) and then packaged into the lentiCRISPR transfer plasmid as previously described by Zhang et al.^46^ For virus production, 293T cells were cotransfected with 5 µg of the lentiCRISPR plasmid library, 4 µg of psPAX2 and 3 μg of pVSVg (Addgene, Watertown, MA, USA) with 30 µl of Lipofectamine™ 3000 transfection reagent (L3000015, Thermo Fisher Scientific). The transfected 293 T cells were incubated at 37 °C, and the transfection medium was replaced after 8 h. Viral particles were harvested 48–72 h after transfection and frozen at −80 °C until further use.

For the in vivo CRISPR-Cas9 knockout screen under oxaliplatin treatment, 4 × 10^7^ DLD1 cells were plated in fifteen 10-cm dishes to ensure sufficient coverage of sgRNAs and infected at a low multiplicity of infection (MOI 0.3) with 10 μg/mL polybrene to ensure that most cells received only one viral construct with high probability. Forty-eight hours after infection, the infected DLD1 cells were selected with 2 μg/ml puromycin for 7 days to select the positively transduced cells and eliminate uninfected cells in order to obtain genome-edited cell pools. The selected cells were subcutaneously injected into the flanks of female BALB/c nude mice (aged 4–5 weeks old, Beijing Vital River Laboratory Animal, Technology, Beijing, China). Once the tumors became palpable, the tumor-bearing mice were randomly assigned into two groups (six mice per group), and treated with PBS or oxaliplatin (7.5 mg/kg) via intraperitoneal *(*i.p.) injection every 3 days. On day 30, we excised and weighed the tumors and then performed genomic DNA extraction using a DNeasy Blood & Tissue Kit (69504, Qiagen, Dusseldorf, Germany). DNA fragments containing the sgRNA sequences were amplified by 2-step PCR using NEBNext Ultra II Q5 Master Mix (M0544L, New England Biolabs, Ipswich, MA, USA) and the primers listed in Supplementary Table S4. The PCR products containing the sgRNA sequences were gel extracted, quantified, mixed, sequenced, and analyzed as previously described.^46^

### Immunohistochemistry (IHC) assay

IHC assays were performed using standard methods as previously reported.^47^ Paraffin-embedded sections were deparaffinized and rehydrated. The endogenous peroxidase activity was blocked with 3% H_2_O_2_ for 10 min. After the addition of the sections to a pressure cooker, antigen was retrieved using sodium citrate buffer or EDTA for 10 min at a sub-boiling temperature. The samples were blocked with 10% FBS for 1 h at room temperature to prevent nonspecific binding, incubated with primary antibody overnight at 4 °C, and then treated with a biotinylated secondary antibody for 1 h at room temperature. The color was developed using a Dako REAL™ EnVision™ Detection System (K5007, Copenhagen, Denmark), and counterstaining was performed using Harris-modified hematoxylin. The stained sections were reviewed and scored independently based on their intensity: 0 (negative), 1 (weak), 2 (moderate), and 3 (strong). The total score was obtained by multiplying the staining intensity scores with the percentage of cells with positive staining. The sections were also stained with hematoxylin and eosin (H&E) according to standard procedures.

### Assay of aconitase activity

The activity of cytosolic aconitase 1 (ACO1) was analyzed with an aconitase activity assay kit (MAK051, Sigma-Aldrich) based on the standard instructions. Briefly, 1 × 10^6^ CRC cells were collected, homogenized in 100 μl of ice-cold assay buffer and centrifuged at 800×*g* and 4 °C for 10 min to collect the supernatant for the ACO1 activity assay. Subsequently, aconitase activation solution was added to activate the sample, and an appropriate reaction mix and developer solution were subsequently added. The absorbance at 450 nm was measured using a Synergy™ Multi-Mode Microplate Reader (Biotek, VT, USA).

### Live/dead cell assay

The LIVE/DEAD Assay Kit (L3224, Thermo Fisher Scientific) was used for the detection of live and dead CRC cells based on the fluorescence microscopy protocol, which can also reflect the viability and cytotoxicity of cells. CRC cells were cultured on 24-well plates and treated with LIVE/DEAD reagents. The labeled cells were viewed under a fluorescence microscope: the dead cells were labeled red, and the live cells were labeled green.

### Assay of cells undergoing apoptosis, necroptosis, pyroptosis, or ferroptosis (fluorescence microscope)

A dead cell detection kit (C1075S, Beyotime, Jiangsu, China) with YO-PRO-1 (YP1) and propidium iodide (PI) staining was used. CRC cells were cultured on 24-well plates and treated with YP1 and PI reagents. The labeled cells were viewed under a fluorescence microscope: the YP1-positive cells (green) were undergoing apoptosis or necroptosis, and the PI-positive cells (red) were undergoing necroptosis, pyroptosis or ferroptosis.

### Apoptosis assay, ROS, and lipid ROS assay (flow cytometry)

Annexin V/PI (KeyGEN, Nanjing, China) was used for the detection of cell apoptosis induced by oxaliplatin. The CM-H2DCF-DA probe (C6827, Thermo Fisher Scientific) and BODIPY™ 581/591 C11 probe (D3861, Thermo Fisher Scientific) were added, and the mixture was incubated in the dark at 37 °C for 30 min to measure the cellular ROS and cellular lipid ROS levels. All these measurements were conducted with a flow cytometer (Beckman Coulter, CA, USA) according to the manufacturer’s instructions. The gating strategy for ROS/lipid ROS analysis is provided in Supplementary Fig. 3f.

### In vivo tumorigenesis and therapeutic study

We constructed cell-derived subcutaneous xenograft (CDX) models and PDX models to evaluate the effects of the combination of NFS1 depletion and oxaliplatin treatment. Female BALB/c nude mice (aged 4–5 weeks, 4–5 mice per group) were obtained from Beijing Vital River Laboratory Animal Technology Co., Ltd. For the CDX models, 2 × 10^6^ NFS1-knockdown or control DLD1 cells and HCT116 cells with NFS1 knockdown and NFS1 overexpression (WT, S293A, and S293D) were injected subcutaneously into the flanks of each mouse, and the mice were then treated with an i.p. injection of PBS or oxaliplatin (7.5 mg/kg every 3 days).

The PDX models were initially generated using fresh tumor samples from two patients with CRC that were subcutaneously implanted into the dorsal flank of mice as the first generation (F0). Once an appropriate volume was reached, the tumors were excised, divided into equal pieces, and subcutaneously implanted into nude mice as the second generation (F1). When the tumors became palpable, the tumor-bearing mice were divided randomly into four groups. NFS1 siRNA or control siRNA (5 nmol per injection, RiboBio) was intratumorally injected into mice receiving oxaliplatin (7.5 mg/kg) or PBS by i.p. injection every 3 days (Fig. 4e). The diameter and width of the tumors from CDX and PDX mice were measured every 3 days and used to calculate the tumor volumes using the formula V = 0.5 × D × W^2^ (V, volume; D, diameter; and W, width). All the mice were sacrificed at the appropriate time, and the tumors were removed, photographed, weighed, and embedded in paraffin for further pathological analysis.

### TdT-mediated dUTP nick-end labeling (TUNEL) assay

The DAB (SA-HRP) TUNEL Cell Apoptosis Detection Kit (G1507, Solarbio Life Science, Beijing, China) was used to perform the TUNEL assay. Briefly, paraffin-embedded sections were deparaffinized, rehydrated, permeabilized, and blocked with 3% H_2_O_2_ for 20 min to block the endogenous peroxidase activity. The sections were incubated with TdT reaction cocktails for 60 min at 37 °C and then treated with streptavidin-HRP reaction mixture for 30 min at 37 °C. Staining was developed using the Dako Detection System, and the images were observed by fluorescence microscopy to count the number of TUNEL-positive cells.

### Immunoprecipitation (IP) assay

Cells were collected, lysed using western and IP lysis buffer (P0013, Beyotime) supplemented with protease inhibitor cocktail (P8430, Sigma-Aldrich) for 30 min on ice, and centrifuged at 12,000 × *g* for 15 min. After protein quantification using a BCA protein assay kit (23225, Thermo Fisher), clarified lysates were incubated with anti-FLAG antibody (1:100) overnight at 4 °C and magnetic protein A/G beads (HY-K0202, MedChemExpress) for 4 h at 4 °C. Normal immunoglobulin (IgG) was used as a negative IP control. The beads were washed at least five times with western and IP lysis buffer, and proteins were eluted by boiling in 1× loading buffer at 100 °C for 15 min and then used for western blotting.

### Luciferase reporter assay

The luciferase activities were detected using the Dual-Luciferase Reporter Assay System according to the manufacturer’s instructions (E1910, Promega). CRC cells were transiently transfected with Renilla (internal control), luciferase reporter plasmids *NFS1* WT or deletion mutant (Mut1, Mut2, and Mut1 + 2) in combination with *MYC* plasmid or the *MYC* siRNA using Lipofectamine™ 3000 transfection reagent (L3000015, Thermo Fisher Scientific). Firefly and Renilla luciferase activities were detected as previously described.^48^

### Chromatin immunoprecipitation (ChIP) assay

ChIP assays were performed using a ChIP kit from Merck Millipore (17–10085, Billerica, MA, USA), as previously described.^48^ The cells were harvested and incubated with 5 μg of anti-MYC antibody or normal control rabbit IgG antibody. The interacting and input DNA were then purified for later analysis. Q-PCR and agarose gel electrophoresis assays were performed to detect the binding regions of the NFS1 promoter that could bind to MYC. The Q-PCR data are presented as percentages of the input. The primers used are listed in Supplementary Table S4.

### Statistical analysis

All experiments were performed at least three times and the results are presented as the mean ± SD. Student’s *t* test was used to compare the differences between two independent groups, analysis of variance (ANOVA) with Dunnett’s/Tukey’s multiple comparisons test was used for comparisons among three or more groups, and Bliss test was used to analyze the synergistic effect between NFS1 suppression and oxaliplatin. Survival curves were assessed using the Kaplan–Meier method (log-rank test). Prognostic factors were assessed via univariate and multivariate Cox regression analyses. Pearson’s correlation analysis and chi-square test were used to investigate the correlations between two continuous variables, and statistical analyses were performed using GraphPad Prism version 8.3.0 (La Jolla, CA, USA) and IBM SPSS Statistics version 21.0 (Armonk, NY, USA). All statistical tests were two-tailed, and *P* values less than 0.05 were considered statistically significant.

## Supplementary information

Supplementary materials

## Data Availability

Analyses of the expression of *NFS1* in multiple human tumors were performed using data obtained from TCGA (http://www.cbioportal.org/publicportal/). The pathway analysis was performed with gene set enrichment analysis (version 4.0.3).^49^ The analysis of the expression of *NFS1* in different types of CRC tissues was supported by the Oncomine database (https://www.oncomine.org/). The correlation between *NFS1* and *MYC, GSS, GCLC, GCLM, GPX2, TXNRD3*, and *CAT* was performed with TIMER (version 2.0) (http://timer.comp-genomics.org/). AnimalTFDB (version 3.0) (http://bioinfo.life.hust.edu.cn/ AnimalTFDB/) was used to predict the MYC-binding sequence. The CRISPR-Cas9 screen-seq and RNA-seq data have been deposited in BioProject (https://www.ncbi.nlm.nih.gov/bioproject) under the accession number PRJNA756841. The data generated in the current study are available within the article and its Supplementary Materials or from the corresponding authors upon reasonable request.
