# Supplementary material for: Phosphorylated NFS1 weakens oxaliplatin-based chemosensitivity of colorectal cancer by preventing PANoptosis
Source: Signal Transduct Target Ther. 2022 Feb 28;7:54. doi: 10.1038/s41392-022-00889-0 (PMC8882671; doi:10.1038/s41392-022-00889-0)
Supplement: Supplementary file 1 — Supplementary materials [file 41392_2022_889_MOESM1_ESM.pdf]

Supplementary Materials for

Phosphorylated NFS1 weakens oxaliplatin-based chemosensitivity of colorectal cancer by preventing PANoptosis

Jin-Fei Lin, Pei-Shan Hu, Yi-Yu Wang, Yue-Tao Tan, Kai Yu, Kun Liao, Qi-Nian Wu, Ting Li, Qi Meng, Jun-Zhong Lin, Ze-Xian Liu, Heng-Ying Pu, Huai-Qiang Ju, Rui-Hua Xu, Miao-Zhen Qiu

Correspondence to: Miao-Zhen Qiu: qiumzh@sysucc.org.cn; Rui-Hua Xu: xurh@sysucc.org.cn; Huai-Qiang Ju: juhq@sysucc.org.cn.

This PDF file includes:

Materials and Methods
Supplementary reference
Supplementary Figures S1 to S6
Supplementary Tables S1 to S6

Materials and Methods

Cell lines

The human embryonic kidney (HEK) 293T, human HCT116, and DLD1 CRC cell lines were purchased from the American Type Culture Collection (ATCC, Rockville, MD, USA). According to standard guidelines, 293T cells were maintained in DMEM and all other cells were maintained in RPMI 1640 (Thermo Fisher Scientific, Carlsbad, CA, USA). All media were supplemented with 10% fetal bovine serum (ExCell Bio, Shanghai, China) and 1% penicillin/streptomycin (WISENT, Nanjing, China). The cells were cultured at 37°C with 5% CO_2. All the cells were authenticated by short tandem repeat (STR) fingerprinting at the Medicine Laboratory of Forensic Medicine Department of Sun Yat-sen University (Guangzhou, China), and tested negative for mycoplasma contamination before use.

Antibodies and reagents

The antibodies used for western blotting were: NFS1 (ab229829, 1:1000), Phospho-MLKL (ab196436, 1:1000), MLKL (ab184718, 1:1000), RIP1 (ab125072, 1:1000), GSDME (ab215191, 1:1000), TFRC (ab214039, 1:1000), FTH1 (ab75972, 1:1000), Phospho-(Serine/Threonine) (ab17464, 1:1000), Phosphothreonine (ab218195, 1:1000), Phosphoserine (ab9332, 1:500) from Abcam (Cambridge, MA, USA); Vinculin (13901S, 1:2000), Caspase-3 (9662S, 1:1000), Cleaved-Caspase-3 (96664S, 1:1000), Caspase-7 (12828S, 1:1000),

Cleaved-Caspase-7 (8438S, 1:1000), Caspase-8 (8592S,1:1000), Caspase-9 (9502P, 1:1000), Cleaved-Caspase-9 (7237S, 1:1000), GSDMD (93709S, 1:1000), Pan-Ubiquitin (3933S, 1:1000), and MYC (18583S, 1:1000) from Cell Signaling Technology (Beverly, MA, USA); Flag-tag (F1804, 1:2000) from Sigma-Aldrich (St. Louis, MO, USA); Phospho-RIP1 (28252-1-AP, 1:1000) from Proteintech (Wuhan, China) and Pan-acetyllysine (PTM-105, 1:1000) from PTM Biolabs (Hangzhou, China). For IHC, NFS1 (sc-365308, 1:100) was purchased from Santa Cruz Biotechnology (Dallas, TX, USA), Ki67 (ZM-0167), and MYC (ZA-0555) from ZSGB-BIO (Beijing, China). Oxaliplatin (S1224) was purchased from Selleck Chemicals (Houston, TX, USA); Z-VAD(OH)-FMK (161401-82-7), 3-methyladenine (5142-23-4), necrostatin-1 (4311-88-0), and disulfiram (T0054) were obtained from TargetMol (Shanghai, China); Ferrostatin-1 (347174-05-4) was procured from MedChemExpress (NJ, USA); N-Acetyl-L-cysteine (NAC, A7250) was procured from Sigma-Aldrich (St. Louis, MO, USA); and glutathione (GSH, S0073) was procured from Beyotime (Jiangsu, China). Ac-DMPD/DMLD-CMK was kindly provided by professor Haiping Hao (China Pharmaceutical University, Nanjing, China)

RNA isolation and Q-PCR assays

Total RNA was extracted with the TRIzol reagent (15596018, Thermo Fisher Scientific) and reverse transcribed to cDNA with a Prime Script RT Master Mix Kit (RR036A, TaKaRa, Tokyo, Japan). The resulting cDNAs were analyzed by Q-PCR using a GoTaq qPCR Master Mix (A6002, Promega) and

a LightCycler 480 instrument (Roche Diagnostics, Switzerland) and the data were normalized using β-Actin, as an endogenous control. Relative gene expression levels were calculated using the $2^{-\Delta\Delta Ct}$ method. The primers used in this study were synthesized by TSINGKE Biological Technology (Guangzhou, China) and are listed in Supplementary Table S4.

Western blotting

Protein was extracted using RIPA buffer (P0013B, Beyotime), separated by SDS-PAGE and transferred to PVDF membranes (IPVH00010, Bio-Rad Laboratories, Hercules, CA, USA). The membranes were blocked (RT, 1 h) and incubated with primary antibodies (4°C, overnight) and then with peroxidase-conjugated secondary antibodies (ZB-2301, ZSGB-BIO) (RT, 1 h). The bands were visualized by a chemiluminescence assay (34096, Thermo Fisher Scientific).

Cell proliferation and cell viability assays

CRC cells were seeded in 96-well plates (NEST Biotechnology, Jiangsu, China) overnight and then treated with different concentrations of oxaliplatin. The proliferation and viability of the cells were analyzed with a CellTiter 96® AQueous One Solution Cell Proliferation Assay (MTS) kit (G3580, Promega) according to the manufacturer's instructions as previously described[47]. The absorbance of 96-well plates at a wavelength of 490 nm was measured using a Synergy™ Multi-Mode Microplate Reader (Biotek).

Colony formation assay

CRC cells were seeded in 6-well plates and incubated for 1 to 2 weeks until obvious clonal spheres had formed. The colonies were washed with PBS, fixed with methanol for 15 min, and then stained with 0.025% crystal violet (C0121, Beyotime) for 15 min. The number of colonies was counted using ImageJ software.

Cell cytotoxicity assay

The culture supernatant of CRC cells was collected and added to 96-well plates. The cell cytotoxicity was evaluated with the Cytotoxicity Detection Kit (11644793001, Roche) to measure the activity of LDH in the cytosol of damaged CRC cells. The absorbance at 490 nm was measured according to standard instructions.

Supplementary reference

1 Ju, H. *et al.* Melatonin overcomes gemcitabine resistance in pancreatic ductal adenocarcinoma by abrogating nuclear factor-κB activation. *J PINEAL RES* **60**, 27-38 (2016).

Supplementary Figure S1

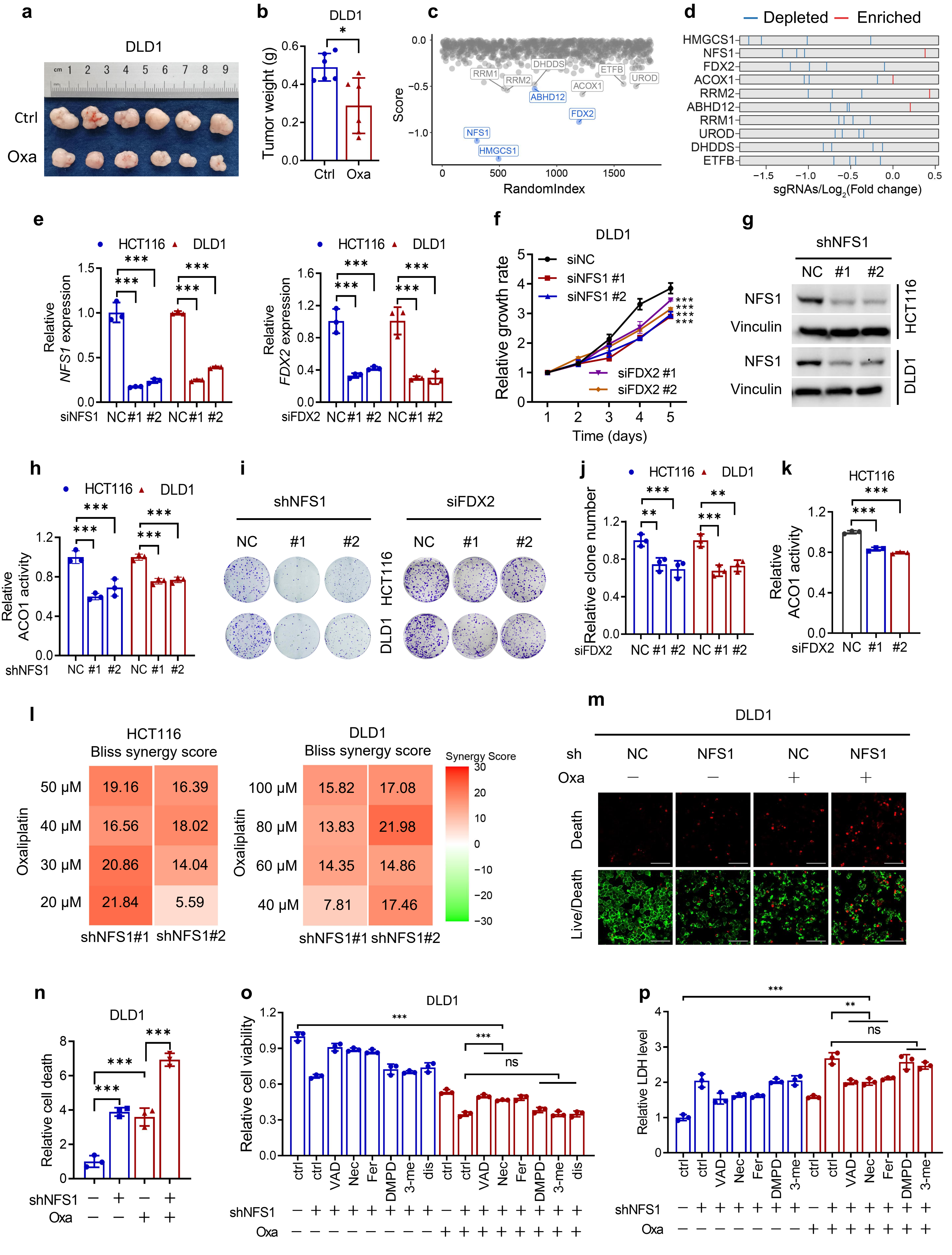

Supplementary Figure S1. *In vivo* CRISPR screening reveals that NFS1 deficiency enhances the sensitivity of CRC cells to oxaliplatin (Oxa).

a. Photograph and comparison of DLD1 CDX tumor size in nude mice after *i.p.* injection with oxaliplatin (7.5 mg/kg) or PBS (n=6). **b**. Statistical analysis of DLD1 CDX tumor weight from (**a**). **c**. Scatter plot showing the genes depleted in the oxaliplatin treatment group compared with the control group. **d**. Detailed information of the four sgRNAs of top 10 candidates depleted in the oxaliplatin treatment group. **e**. Q-PCR detection of *NFS1* or *FDX2* mRNA expression in *NFS1*-knockdown and control HCT116 and DLD1 cells. **f**. MTS analysis of the cell proliferation of DLD1 cells with NFS1 or FDX2 silence. **g**. Western blotting analysis of NFS1 protein expression in *NFS1*-knockdown and control HCT116 and DLD1 cells. **h.** Activity analysis of cytosolic ACO1 of control and *NFS1*-knockdown HCT116 and DLD1 cells. **i.** Colony formation analysis reflected the proliferation of control and *NFS1*-knockdown or *FDX2*-knockdown HCT116 and DLD1 cells. **j.** Quantification of colony formation of control and FDX2-knockdown HCT116 and DLD1 cells. **k.** ACO1 Activity analysis of control and *FDX2*-knockdown HCT116 cells. **l.** Bliss test for Fig. 1g (left) and 1h (right) via Synergy Finder web (http://www.synergyfinder.org/). Bliss synergy score > 10 suggests synergistic effect. **m**. The live/dead viability/cytotoxicity assay showing dead (red) and live (green) cells from the control and *NFS1*-knockdown groups treated with 80 μM oxaliplatin for 24 h. Scale bar=100 μm. **n**. Quantification of relative dead cell from (**m**). **o**, **p**. Cell viability **(o)** and cytotoxicity **(p)** of control and *NFS1*-knockdown DLD1 cotreated with or without 80 μm oxaliplatin and the apoptosis inhibitor Z-VAD-FMK (VAD, 25 μM), the necrosis inhibitor necrostatin (Nec, 20 μM), the ferroptosis inhibitor Ferrostatin-1 (Fer, 10 μM), the pyroptosis inhibitors Ac-DMPD-CMK (DMPD, 20 μM) and disulfiram (dis, 1 μM) or the autophagy inhibitor 3-methyladenine (3-me, 10 μM). Vinculin was included as a loading control. The data in **b** are representative of six independent experiments and those in **e**, **f, h**, **j**, **k** and **n**-**p** are representative of three independent experiments, and all are presented as the mean±S.D. The *P* value in **b** was calculated by two-tailed unpaired Student's t test, those in **e**, **f**, **h** and **j** were calculated by two-way ANOVA with Dunnett's multiple comparisons test and those in **k, n-p** were calculated by one-way ANOVA with Tukey's multiple comparisons test. *$P < 0.05$; **$P < 0.01$; ***$P < 0.001$.

Supplementary Figure S2

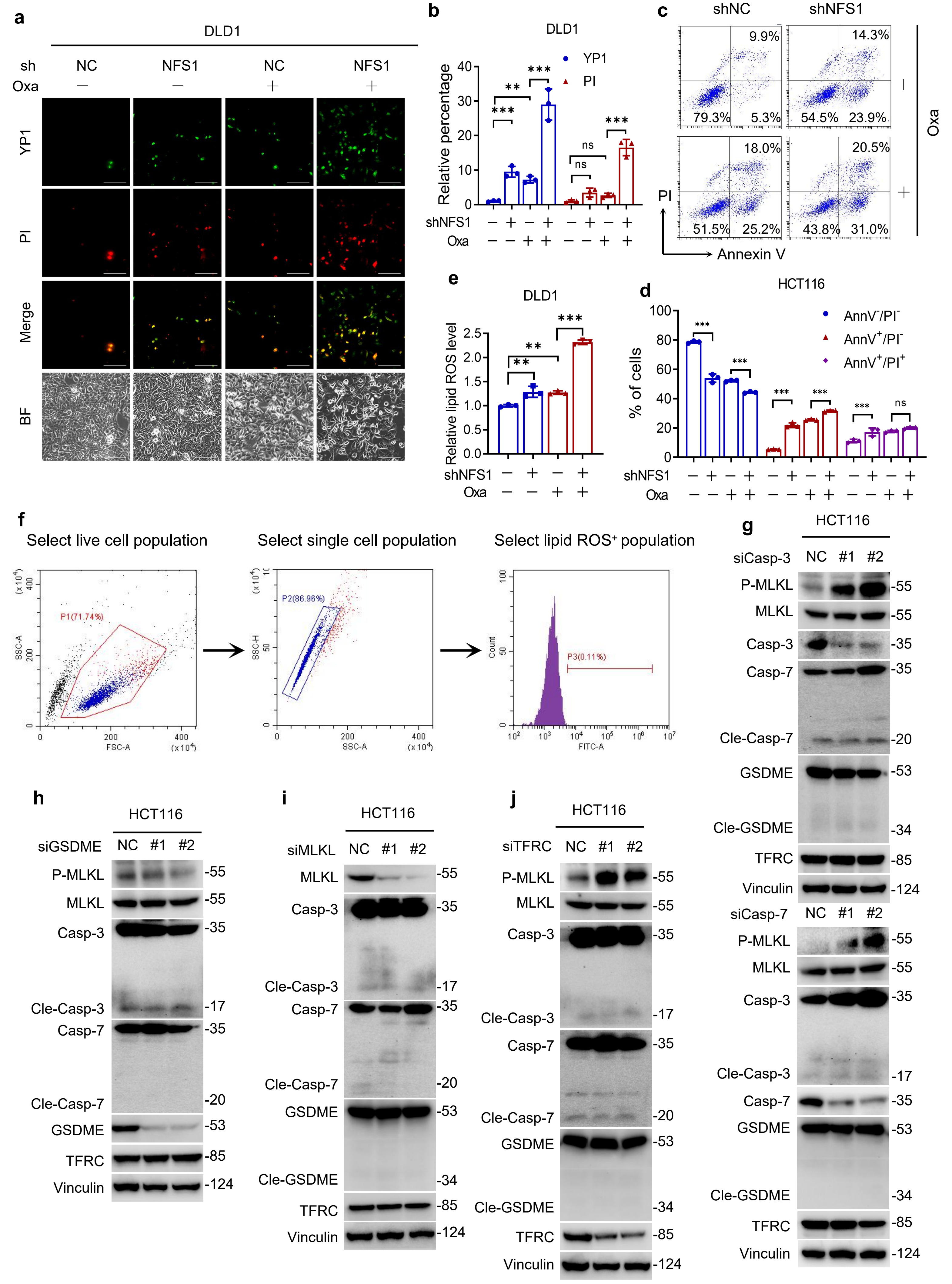

Supplementary Figure S2. NFS1 deficiency synergizes with oxaliplatin treatment to induce PANoptosis.

a. Representative images showing YP1$^+$ cells (green) which may undergo apoptosis or necroptosis and PI$^+$ cells (red) which may undergo apoptosis, necroptosis, pyroptosis or ferroptosis within control and *NFS1*-knockdown DLD1 cells treated with 80 μM oxaliplatin for 24 h. The bottom panel shows representative bright fields. Scale bar=100 μm. **b.** Quantification of the YP1$^+$ and PI$^+$ apoptotic and necrotic cells from **(a)**. **c**. Flow cytometry analysis with Annexin V/PI staining estimating the percentages of live cells (Annexin V$^-$/PI-), early apoptotic cells (Annexin V$^+$/PI$^-$) and late apoptotic cells (Annexin V$^+$/PI$^+$) in control and *NFS1*-knockdown HCT116 cells treated with PBS or oxaliplatin (40 μM, 24 h). **d**. Quantification of the live cells (Annexin V$^-$/PI$^-$), early apoptotic cells (Annexin V$^+$/PI$^-$) and late apoptotic cells (Annexin V$^+$/PI$^+$) from (**c**). **e.** Lipid ROS was assessed with the BODIPY™ 581/591 C11 probe assay in control and *NFS1*-knockdown DLD1 cells treated with oxaliplatin (80 μM, 24 h). **f**. The gating strategy used for ROS/lipid ROS analysis in this study. **g-j.** Western blotting analysis of phosphorylated MLKL, total MLKL, caspase-3, cleaved caspase-3, caspase-7, cleaved caspase-7, GSDME, cleaved GSDME and TFRC expression in HCT116 cells after silencing *caspase-3* **(g)**, *caspase-7* **(g)**, *GSDME* **(h)**, *MLKL* **(i)**, *TFRC* **(j).** Vinculin was included as a loading control. The data in **b**, **d** and **e** are representative of three independent experiments and presented as the mean±S.D. The *P* values in **b** and **d** were calculated by two-way ANOVA and this in **e** was calculated by one-way ANOVA with Tukey's multiple comparisons test. $^{**}P < 0.01$, $^{***}P < 0.001$.

Supplementary Figure S3

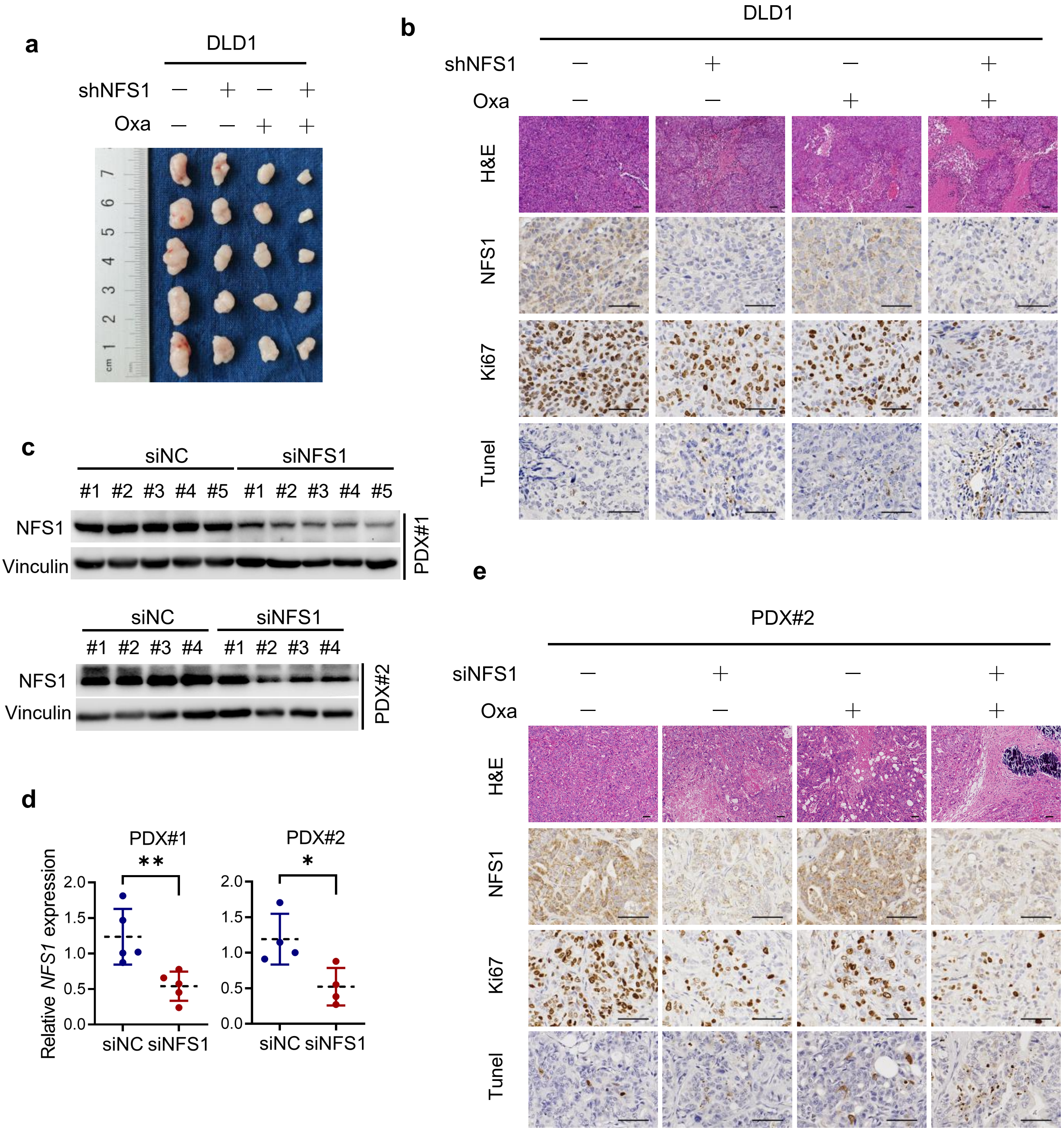

Supplementary Figure S3. NFS1 deficiency enhances the antitumor effect of oxaliplatin *in vivo*.

a. Photograph and comparison of the sizes of excised tumors from DLD1-based xenografts (n=5). **b**, **e**. Representative H&E and IHC staining images of NFS1, Ki67 and TUNEL in DLD1-based (**b**) and PDX #2-based (**e**) paraffin-embedded subcutaneous tumor sections. Scale bar=50 μm. **c, d,** Western blottingting (**c**) and Q-PCR(**d**) analysis of NFS1 expression in tumor tissues from PDX models (Fig. **5e**). Vinculin was included as a loading control.

The data in **d** are representative of five (left) and four (right) independent experiments and presented as the mean±S.D. The *P* values in **d** were calculated by two-tailed unpaired Student's t test, $*P < 0.05$, $**P < 0.01$.

Supplementary Figure S4

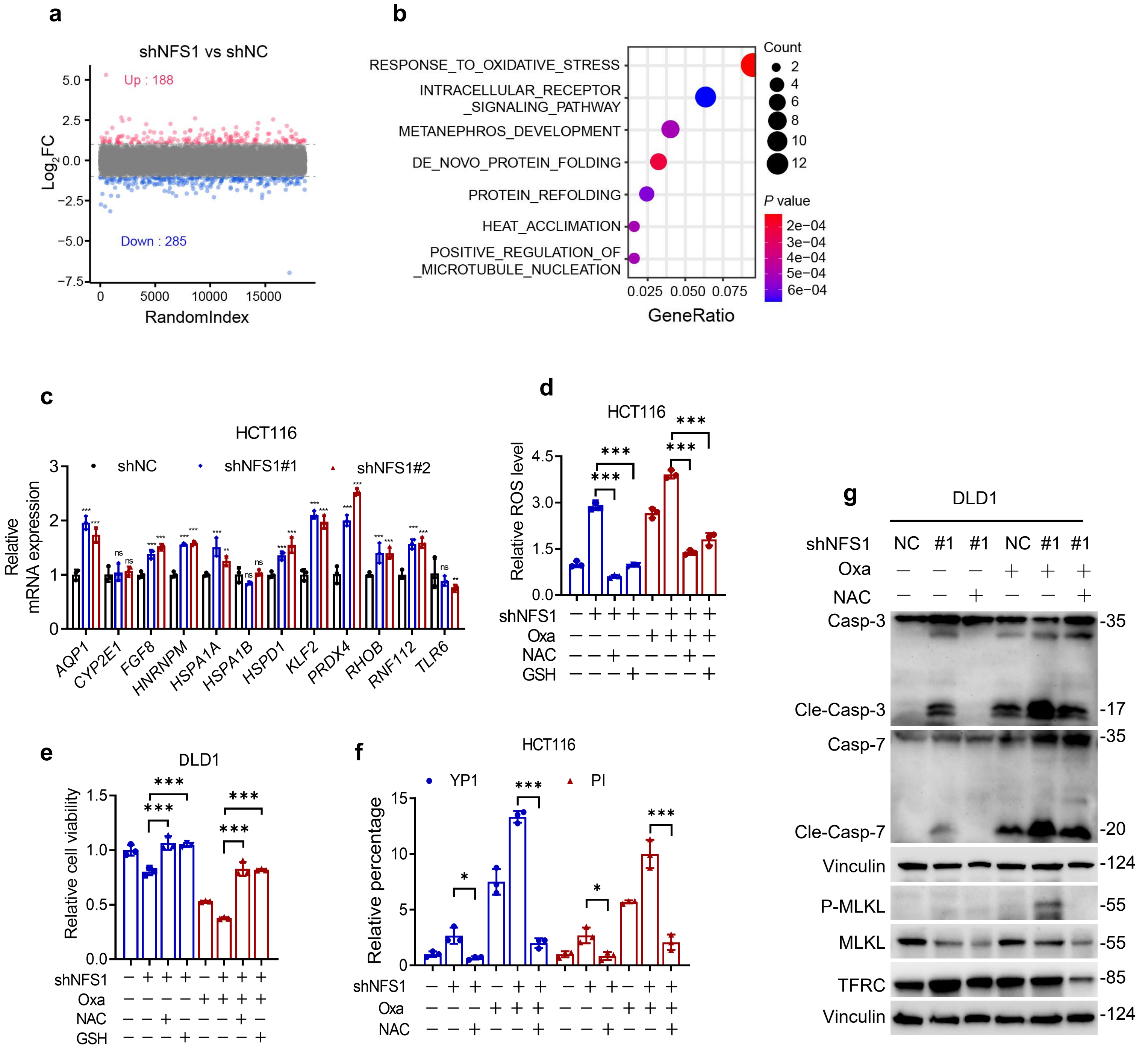

Supplementary Figure S4. Oxidative stress is critical for NFS1 deficiency-induced PANoptosis under oxaliplatin treatment.

a. Scatter plot showing the upregulated and downregulated genes in the *NFS1*-knockdown group compared with the control group under oxaliplatin treatment (40 μM, 24 h) based on RNA-seq, and genes with twofold change are highlighted. **b**. Scatter grams of the upregulated pathways from the genes with twofold upregulation in *NFS1*-knockdown group compared with control group based on GO enrichment analysis. The analyzed RNA-seq data was provided in Supplementary Table S2. **c.** Q-PCR detection of *AQP1, CYP2E1, FGF8, HNRNPM, HSPA1A, HSPA1B, HSPD1, KLF2, PRDX4, RHOB, RNF112,* and *TLR6* mRNA expression from **(b)** which enriched in “response to oxidative stress” pathway in HCT116 cells under oxaliplatin treatment (40 μM, 24 h). **d**. The ROS analysis of HCT116 cells treated with 40 μM oxaliplatin combined with 5 mM NAC or 5 mM GSH for 24 h after *NFS1*-knockdown. **e**. Cell viability of DLD1 cells treated with 80 μM oxaliplatin combined with 5 mM NAC or 5 mM GSH for 24 h after *NFS1* knockdown. **f**. Quantification of $YP1^+$ and PI^+ HCT116 cells from Fig. 4e. **g**. Western blotting analysis of caspase-3, cleaved caspase-3, caspase-7, cleaved caspase-7, phosphorylated MLKL, total MLKL and TFRC expression in control and *NFS1*-knockdown DLD1 cells after treatment of 80 μM oxaliplatin combined with 5 mM NAC (24 h). Vinculin was included as a loading control. The data in **c-f** are representative of three independent experiments and presented as the mean±S.D. The *P* values in **d** and **e** were calculated by one-way ANOVA, and those in **c** and **f** were calculated by two-way ANOVA with Tukey’s multiple comparisons test. *$P < 0.05$, **$P < 0.01$, ***$P < 0.001$.

Supplementary Figure S5

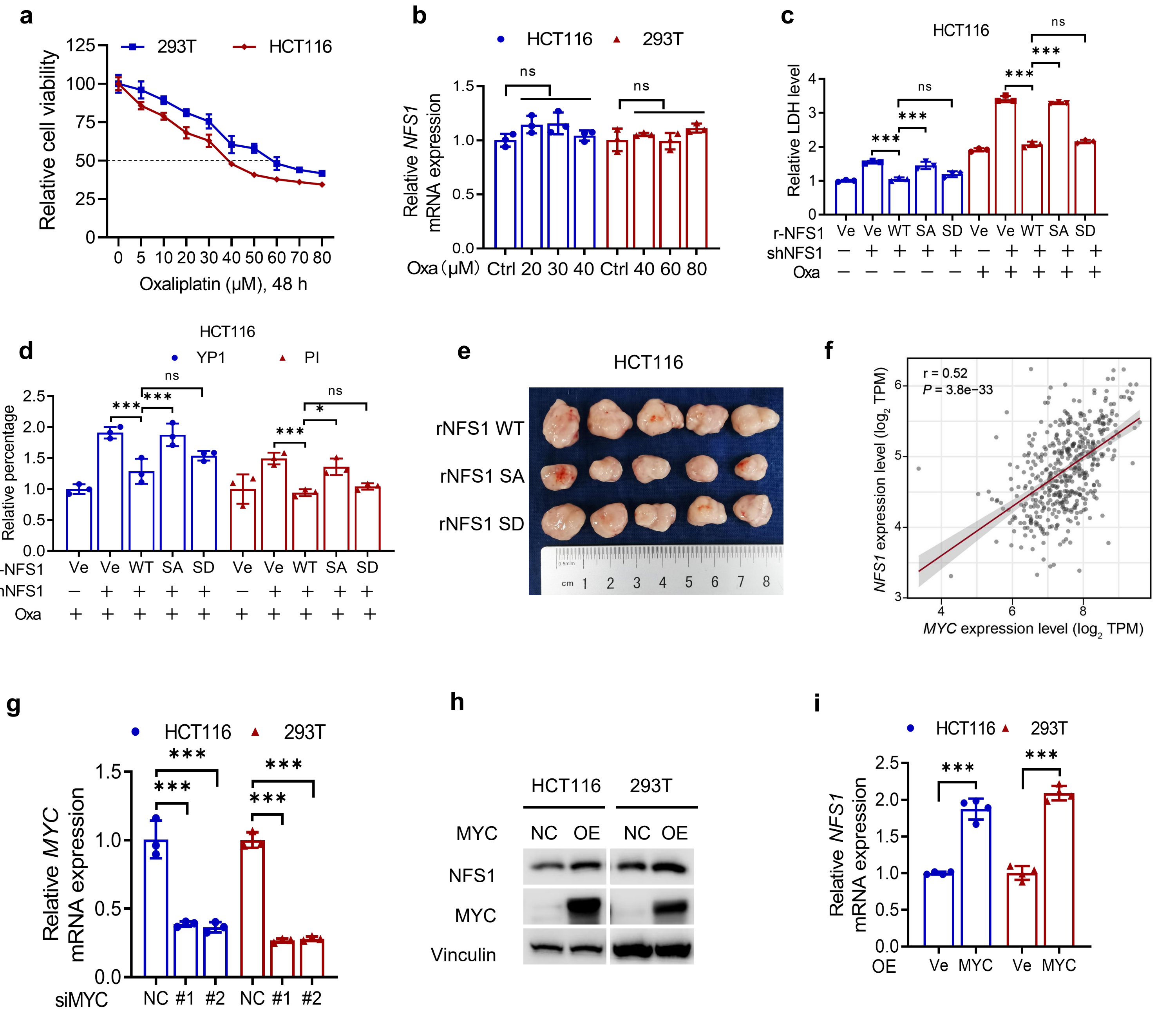

Supplementary Figure S5. *NFS1* is transcriptionally regulated by MYC.

a. Cell viability of HCT116 and 293T cells treated with different concentrations of oxaliplatin for 48 h, showing the different sensitivity to oxaliplatin. **b**. Q-PCR detection of *NFS1* mRNA levels in HCT116 and 293T cells treated with different concentrations of oxaliplatin for 24 h. **c.** Cell cytotoxicity assay in HCT116 cells overexpressing *rNFS1* WT and S293A or S293D mutant with or without oxaliplatin treatment (40 μM, 24 h). **d.** Quantification of $YP1^{+}$ and PI^{+} HCT116 cells from Fig. **5 i**. **e.** Photograph showing the comparison of CDX tumor size after implantation of *NFS1*-knockdown HCT116 cells overexpressing *rNFS1* WT or S293A or S293D mutant, followed by *i.p.* injection of oxaliplatin (7.5 mg/kg) (n =5). **f**. Correlations between *NFS1* mRNA expression and *MYC* mRNA expression from the TCGA database (n=458) (http://timer.comp-genomics.org/). **g**. Q-PCR analysis of *MYC* in HCT116 and 293T cells with MYC downregulation or control. **h**, **i**. Western blottingting **(h)** and Q-PCR **(i)** analysis of NFS1 expression in HCT116 and 293T cells with or without upregulated MYC. Vinculin was included as a loading control. The data in **a**-**d, g** and **i** are representative of three independent experiments and presented as the mean±S.D. The *P* values in **a, b** and **g** were calculated by two-way ANOVA with Dunnett's multiple comparisons test, those in **c** were calculated by one-way ANOVA with Tukey's multiple comparisons test, those in **d** were calculated by two-way ANOVA with Tukey's multiple comparisons test, and those in **i** were calculated by two-tailed unpaired Student's t test. *$P < 0.05$, ***$P < 0.001$.

Supplementary Figure S6

a

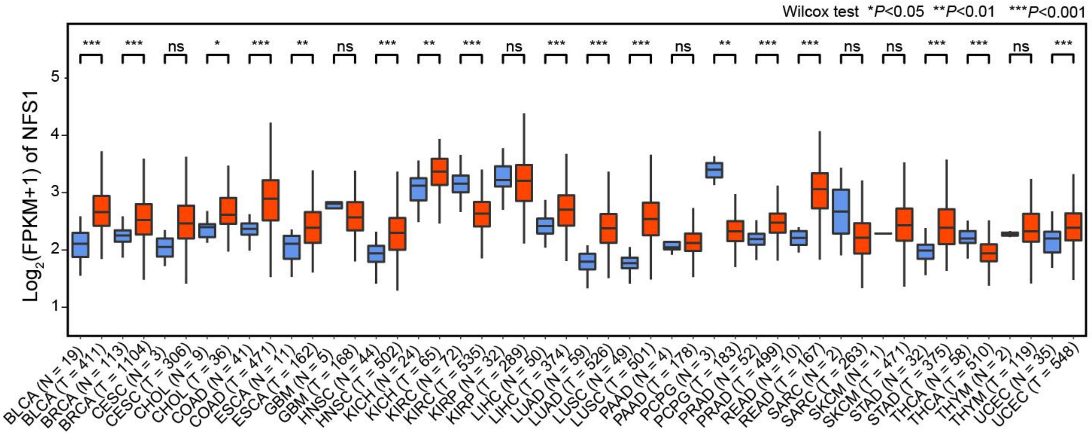

b

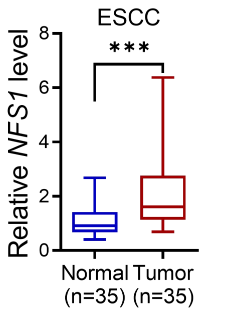

c

Table 1. The relative mRNA levels of *NFS1* in CRC tissues compared with normal tissues

Types of CRC VS. Normal tissues	Fold change	P value	t-test	Reference or Source
Rectal adenocarcinoma	1.837	1.52E-14	9.53	TCGA colorectal
Colon adenocarcinoma	1.613	8.67E-12	8.55	TCGA colorectal
Rectal adenocarcinoma	1.583	2.38E-28	16.03	TCGA colorectal 2
Rectosigmoid adenocarcinoma	1.574	2.54E-10	10.21	TCGA colorectal 2
Colon carcinoma	1.949	2.28E-11	20.48	Skrzypczak colorectal 2
Colon adenoma	1.817	8.95E-5	8.60	Skrzypczak colorectal 2
Colon carcinoma	1.917	8.75E-10	8.35	Hong colorectal
Rectal adenocarcinoma	1.819	5.52E-20	11.89	Gaedcke colorectal
Rectosigmoid adenocarcinoma	1.556	6.89E-4	4.28	Kaiser colon

d

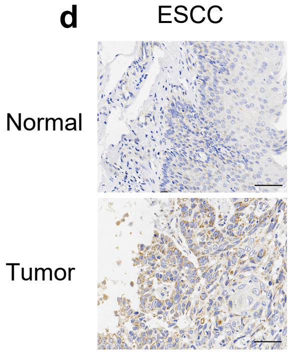

e

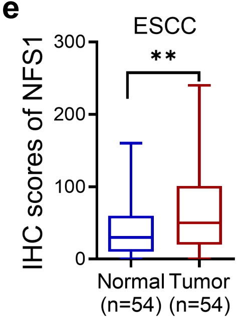

f

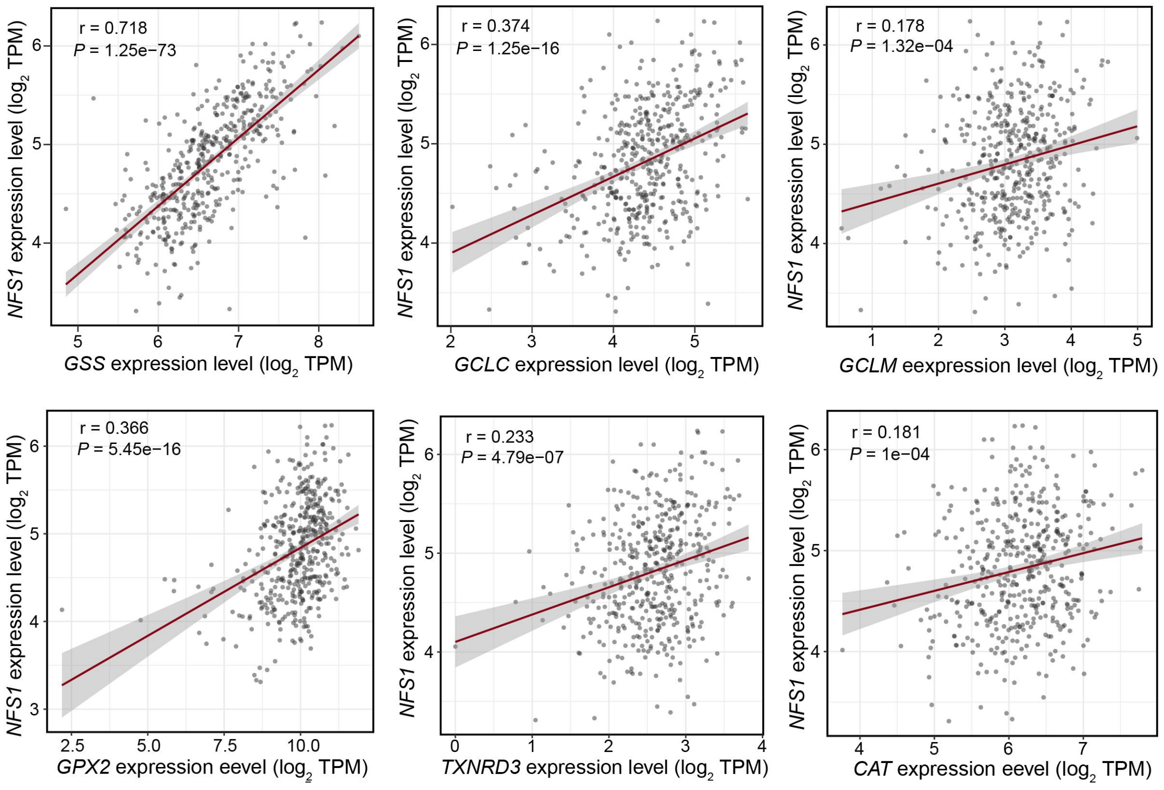

Supplementary Figure S6. NFS1 is highly expressed in tumor tissues.

a. Analysis of NFS1 expression at the transcription level in multiple human cancers from the TCGA database, including colon adenocarcinoma (COAD). **b.** Q-PCR detection of *NFS1* mRNA expression in 35 pairs of ESCC tumor tissues (T) and adjacent normal tissues (N). **c**. The relative mRNA levels of *NFS1* in different types of CRC tissues compared with normal tissues from the Oncomine database. **d, e.** Representative IHC staining images of NFS1 （Scale bar=100 μm) **(d)** and the IHC staining scores **(e)** of NFS1 expression in paired tumor tissues (T) and adjacent normal tissues (N) from ESCC patients (n=54). **f**. The TCGA database showing a positively correlation between *NFS1* expression and *GSS, GCLC, GCLM, GPX2, TXNRD3* and *CAT* expression (n=458). The data in **a, b** and **e** are presented in the form of box and whisker plots (minimum-maximum) with the horizontal line in each box representing the median, The *P* values in **b** and **e** were calculated by two-tailed paired Student's t test. *P < 0.05, **P < 0.01, ***P < 0.001.

Supplementary Table S1: Correlation analysis for clinicopathologic variables in NFS1 expression among 371 CRC patients

Variable	Low NFS1<br>n (%)	High NFS1<br>n (%)	*P* value
Total	185 (49.9)	186 (50.1)	
Age, years			0.049
< 57	104 (56.2)	85 (45.7)	
≥ 57	81 (43.8)	101 (54.3)	
Gender			0.916
Male	107 (57.8)	109 (58.6)	
Female	78 (42.2)	77 (41.4)	
Clinical stage			0.104
I-II	73 (39.5)	58 (31.2)	
III-IV	112 (60.5)	128 (38.8)	
Degree of differentiation			0.021
Well/moderate	138 (74.6)	157 (84.4)	
Poor and others	47 (25.4)	29 (15.6)	
Tumor depth			0.274
m/sm/mp	19 (10.3)	13 (7.0)	
ss/se/si	166(89.7)	173(93.0)	
Vascular invasion			0.822
Absent	130 (70.3)	128(68.8)	
Present	55 (29.7)	58 (31.2)	
Perineural invasion			0.062
Absent	100 (54.1)	82 (44.1)	
Present	85(45.9)	104 (55.9)	

The *P* value was determined by a Chi-square test. All the statistical tests were two-sided. Abbreviations: m: tumor invasion of mucosa; sm: submucosa; mp: muscularis propria; ss:subserosa; se: serosa penetration; si: invasion to adjacent structures.

Supplementary Table S2: Univariate and multivariate analysis of prognostic factors for overall survival among 371 CRC patients.

Factors	Univariate		Multivariate*	
	HR (95% CI)	*P* value	HR (95% CI)	*P* value
Age, years (≤ 60 vs > 60)	1.497 (0.947-2.365)	0.084	1.796 (1.067-3.025)	0.028
Gender (Male vs Female)	0.635 (0.395-1.019)	0.060	-	-
Clinical stage (I-IIvs III-IV)	5.446 (2.902-10.221)	0.000	2.574(1.280-5.175)	0.008
Degree of differentiation (Well, moderate vs Poor and others)	1.983(1.167-3.371)	0.011	1.941(1.061-3.551)	0.031
Tumor depth (m, sm, mp vs ss, se, si)	1.168 (0.507-2.691)	0.715	-	-
Vascular invasion (absent vs present)	5.660(3.455-9.273)	0.000	3.187(1.701-5.970)	0.000
Perineural invasion (absent vs present)	4.125 (2.486-6.845)	0.000	-	-
NFS1 expression (low vs high)	1.662 (1.048-2.633)	0.031	1.669 (0.990-2.814)	0.055

The *P* value was determined by the univariate and multivariate cox regression analysis. Al the statistical tests were two-sided. Abbreviations: CRC: Colorectal cancer; m: tumor invasior of mucosa; sm: submocosa; mp: muscular is propria; ss:subserose; se: serosa penetration si: invasion to adjacent structures. HR: hazard ratio; CI: confidence interval.

* For the multivariate model, HR and *P* values were shown by backward method only.

Supplementary Table S3: Targeted sequences of siRNAs and shRNAs

Gene names	Targeted sequences
si *NFS1* #1	GCATGTGAAGCGTCTTCGA
si *NFS1* #2	GAGCAATTGGCACTGATGA
si *NFS1* (*in vivo*)	GCATGTGAAGCGTCTTCGA
si *FDX2* #1	GGAAGACGACATGCTAGAC
si *FDX2* #2	AACGTGGTGTTCGTAGACC
si *MYC* #1	AGACCUUCAUCAAAAACAU
si *MYC* #2	GAGCUAAAACGGAGCUUUU
sh *NFS1* #1	GCAAGTAGCATCTCTGATT
sh *NFS1* #2	GGTTTGGAATTGGCCGCTT
si *caspase-3* #1	GGATCGTTGTAGAAGTCTA
si *caspase-3* #2	GACGCTACTTTTCATGCAA
si *caspase-7* #1	GCACGGAAAAGACCTGGAA
si *caspase-7* #2	GGACCGAGTGCCTACATAT
si *MLKL* #1	CTGGAGATATCCCGTTTCA
si *MLKL* #2	CGCTGTTACTTCAGGTTGA
si *GSDME* #1	CCCACTGCTTCTTTGTATA
si *GSDME* #2	CAAGCAGCTGTTTATGACA
si *TFRC* #1	GTAGGATGGTAACCTCAGA
si *TFRC* #2	GCACAGCTCTCCTATTGAA

Supplementary Table S4: Sequence of primers used in PCR analysis

Name	Forward primer (5'-3')	Reverse primer (5'-3')
β-Actin	CATGTACGTTGCTATCCAGGC	CTCCTTAATGTCACGCACGAT
NFS1	TGGATGTGCAAGCTACAACTC	GATCAGCTCCAATCAGAGATGC
FDX2	TTGAGGGAATAGCCAGGTGC	ATGGGACTCTCTCCCAAGCA
MYC	GTCAAGAGGCGAACACACAAC	TTGGACGGACAGGATGTATGC
Screen first PCR	AATGGACTATCATATGCTTACCGTAACTTGAAAGTATTTCG	TCTACTATTCTTTCCCCTGCACTGTTGTGGGCGATGTGCGCTCTG
Screen second PCR-1	AATGATACGGCGACCACCGAGATCTACACTCTTTCCCTACACGACGCTCTTCCGATCTTAAGTAGAGTCTTGTGGAAAGGACGAAACACCG	CAAGCAGAAGACGGCATACGAGATAAGTAGAGGTGACTGGAGTTCAGACGTGTGCTCTTCCGATCTTTCTACTATTCTTTCCCCTGCACTGT
Screen second PCR-2	AATGATACGGCGACCACCGAGATCTACACTCTTTCCCTACACGACGCTCTTCCGATCTATACACGATCTCTTGTGGAAAGGACGAAACACCG	CAAGCAGAAGACGGCATACGAGATACACGATCGTGACTGGAGTTCAGACGTGTGCTCTTCCGATCTATTCTACTATTCTTTCCCCTGCACTGT
Screen second PCR-3	AATGATACGGCGACCACCGAGATCTACACTCTTTCCCTACACGACGCTCTTCCGATCTGATCGCGCGGTTCTTGTGGAAAGGACGAAACACCG	CAAGCAGAAGACGGCATACGAGATCGCGCGGTGTGACTGGAGTTCAGACGTGTGCTCTTCCGATCTGATTCTACTATTCTTTCCCCTGCACTGT
Screen second PCR-4	AATGATACGGCGACCACCGAGATCTACACTCTTTCCCTACACGACGCTCTTCCGATCTCGATCATGATCGTCTTGTGGAAAGGACGAAACACCG	CAAGCAGAAGACGGCATACGAGATCATGATCGGTGACTGGAGTTCAGACGTGTGCTCTTCCGATCTCGATTCTACTATTCTTTCCCCTGCACTGT
Screen second PCR-5	AATGATACGGCGACCACCGAGATCTACACTCTTTCCCTACACGACGCTCTTCCGATCTTCGATCGTTACCATCTTGTGGAAAGGACGAAACACCG	CAAGCAGAAGACGGCATACGAGATCGTTACCAGTGACTGGAGTTCAGACGTGTGCTCTTCCGATCTTCGATTCTACTATTCTTTCCCCTGCACTGT
Screen second PCR-6	AATGATACGGCGACCACCGAGATCTACACTCTTTCCCTACACGACGCTCTTCCGATCTATCGATTCCTTGGTTCTTGTGGAAAGGACGAAACACCG	CAAGCAGAAGACGGCATACGAGATTCCTTGGTGTGACTGGAGTTCAGACGTGTGCTCTTCCGATCTATCGATTCTACTATTCTTTCCCCTGCACTGT
NFS1 promoter-1	TGCCACTGCACTCCAACC	CTTTGCCAAGGATCTTCTGCAC

NFS1 promoter-2	GGCAAAGCTGGATAATAGGCTG	CCTCCTCAGGATCGGAATGC
NFS1 promoter-3	CCGATCCTGAGGAGGAAACC	CTTAAACTGCAGGCAACACCAG
NFS1 promoter-4	GGTGTTGCCTGCAGTTTAAGAC	GGAAAGAAACTCGTTCAGCGG
NFS1 promoter-5	CGAGTTTCTTTCCTTATGCGCA	GTCAGGTTGGAAGCGTCAG
NFS1 promoter-6	TCAGGCTCCAGGCCGAATG	GAACACAGCCTCCTTCTTTCG
NFS1 promoter-7	CGAAGCCCATTTTGACACGG	GCGAAGAGGGTGGTGGAG
NFS1 promoter-8	CACCACCCTCTTCGCGTT	GCCTCCAAGCGGCTCGGA
AQP1	CTGGGCATCGAGATCATCGG	ATCCCACAGCCAGTGTAGTCA
CYP2E1	GTGATGCACGGCTACAAGG	GGGTGGTCAGGGAAAACCG
FGF8	GACCCCTTCGCAAAGCTCAT	CCGTTGCTCTTGGCGATCA
HNRNPM	CTCTTAATGGACGCTGAAGGAAA	CGCTCAGACTATGCTTGTTTAGG
HSPA1A	AGCTGGAGCAGGTGTGTAAC	CAGCAATCTTGGAAAGGCCC
HSPA1B	TCAGGCCCTACCATTGAGGA	CCTTGAGTCCCAACAGTCCA
HSPD1	CTACTGTACTGGCACGCTCTA	CAACAGCTAACATCACACCTCTC
KLF2	CTACACCAAGAGTTCGCATCTG	CCGTGTGCTTTCGGTAGTG
PRDX4	AGAGGAGTGCCACTTCTACG	GGAAATCTTCGCTTTGCTTAGGT
RHOB	CAGTAAGGACGAGTTCCCCG	GTCCACCGAGAAGCACATGA
RNF112	GCCACGACTTCTGCATACG	TCTTCTGCTTGCATATCTTCCG
TLR6	TTCTCCGACGGAAATGAATTTGC	CAGCGGTAGGTCTTTTGGAAC

Supplementary Table S5. The top 50 candidates depleted in the oxaliplatin treatment group compared with control group (CRISPR screen)

Number	ID	Score	FDR
1	HMGCS1	-1.2849	0.02104
2	NFS1	-1.0917	0.002475
3	FDX2	-0.88582	0.002475
4	ACOX1	-0.58671	0.192169
5	RRM2	-0.54296	0.121287
6	ABHD12	-0.52861	0.022772
7	RRM1	-0.52533	0.514321
8	UROD	-0.50055	0.159901
9	DHDDS	-0.47997	0.388759
10	ETFB	-0.47566	0.286068
11	MAT2A	-0.45773	0.286068
12	DUT	-0.39418	0.02104
13	NUDT4	-0.37927	0.507426
14	PGAM1	-0.3603	0.153465
15	SCD	-0.34729	0.999976
16	PLA2G10	-0.33296	0.286068
17	AGPAT2	-0.3328	0.159901
18	ACBD3	-0.32924	0.622069
19	GAL3ST3	-0.32209	0.473597
20	B3GALT6	-0.31363	0.159901
21	ILVBL	-0.30932	0.677523
22	NUDT19	-0.30799	0.444719
23	ENPP7	-0.30668	0.639765
24	PLA2G2F	-0.30334	0.634253
25	PLPPR2	-0.30209	0.473597
26	CERK	-0.30097	0.999976
27	APRT	-0.29969	0.388759
28	HS3ST3A1	-0.29867	0.315842
29	NDUFAF2	-0.29519	0.999976
30	PTEN	-0.29471	0.677523

31	ACLY	-0.2908	0.699805
32	DPM1	-0.28455	0.499775
33	AMY2A	-0.28334	0.847306
34	PI4KA	-0.28281	0.999976
35	MMAB	-0.27919	0.677523
36	UQCRH	-0.27801	0.473597
37	ADCY1	-0.27629	0.677523
38	COMT	-0.27451	0.507426
39	THNSL2	-0.27398	0.572365
40	FASN	-0.27265	0.566907
41	COX4I2	-0.27082	0.870797
42	MPO	-0.26722	0.999976
43	GGT6	-0.26604	0.928318
44	NUDT21	-0.25965	0.999976
45	PTGIS	-0.25853	0.507426
46	THEM5	-0.25843	0.999976
47	ENO3	-0.25653	0.677523
48	ACSM4	-0.25614	0.804419
49	ACSF3	-0.25571	0.605473
50	ETFDH	-0.25463	0.999976

Supplementary Table S6: Gene Onotology enrichment analysis of genes upregulated twofold in the *NFS1*-knockdown group compared with control group (top 50)

Description	GeneRatio	BgRatio	pvalue	p.adjust	qvalue	geneID	Count
GO_RESPONSE_TO_OXIDATIVE_STRESS	12/129	458/17901	0.0001	0.1676	0.1547	AQP1/CYP2E1/FGF8/HNRNPM/HSPA1A/HSPA1B/HSPD1/KLF2/PRDX4/RHOB/RNF112/TLR6	12
GO_DE_NOVO_PROTEIN_FOLDING	4/129	41/17901	0.0002	0.1676	0.1547	DNAJB13/HSPA1A/HSPA1B/HSPD1	4
GO_HEAT_ACCLIMATION	2/129	5/17901	0.0005	0.1676	0.1547	HSPA1A/HSPA1B	2
GO_POSITIVE_REGULATION_OF_MICROTUBULE_NUCLEATION	2/129	5/17901	0.0005	0.1676	0.1547	HSPA1A/HSPA1B	2
GO_METANEPHROS_DEVELOPMENT	5/129	91/17901	0.0005	0.1676	0.1547	FGF8/GDF11/NKX3-1/OSR2/WNT4	5
GO_PROTEIN_REFOLDING	3/129	23/17901	0.0006	0.1676	0.1547	HSPA1A/HSPA1B/HSPD1	3
GO_INTRACELLULAR_RECEPTOR_SIGNALING_PATHWAY	8/129	265/17901	0.0007	0.1676	0.1547	CYP27B1/HSPA1A/HSPA1B/KLF2/NKX3-1/NR6A1/PAGR1/ZCCHC3	8
GO_REGULATION_OF_NUCLEOTIDE_BINDING_OLIGOMERIZATION_DOMAIN_CONTAINING_2_SIGNALING_PATHWAY	2/129	6/17901	0.0008	0.1676	0.1547	HSPA1A/HSPA1B	2
GO_CHAPERONE_MEDIATED_PROTEIN_FOLDING	4/129	60/17901	0.0009	0.1676	0.1547	DNAJB13/FKBP5/HSPA1A/HSPA1B	4
GO_REGULATION_OF_MICROTUBULE_NUCLEATION	2/129	7/17901	0.0011	0.1676	0.1547	HSPA1A/HSPA1B	2
GO_TOLL_SIGNALING_PATHWAY	2/129	7/17901	0.0011	0.1676	0.1547	PALM3/PELI2	2
GO_EMBRYONIC_HINDLIMB_MORPHOGENESIS	3/129	29/17901	0.0012	0.1676	0.1547	FGF8/OSR2/PITX1	3
GO_NEGATIVE_REGULATION_OF_STEROID_METABOLIC_PROCESS	3/129	30/17901	0.0013	0.1676	0.1547	CYP27B1/ERLIN2/WNT4	3
GO_PROTEIN_FOLDING	7/129	230/17901	0.0014	0.1676	0.1547	CCT5/DNAJB13/FKBP5/HSPA1A/HSPA1B/HSPD1/PRDX4	7
GO_NEUROENDOCRINE_CELL_DIFFERENTIATION	2/129	8/17901	0.0014	0.1676	0.1547	FGF8/WNT4	2
GO_CHAPERONE_COFACTOR_DEPENDENT_PROTEIN_REFOLDING	3/129	32/17901	0.0016	0.1744	0.1610	DNAJB13/HSPA1A/HSPA1B	3
GO_POSITIVE_REGULATION_OF_MICROTUBULE_POLYMERIZATION_OR_DEPOLYMERIZATION	3/129	34/17901	0.0019	0.1960	0.1809	HSPA1A/HSPA1B/MAPRE1	3
GO_REGULATION_OF_NUCLEOTIDE_BINDING_OLIGOMERIZATION_D	2/129	10/17901	0.0022	0.2029	0.1873	HSPA1A/HSPA1B	2

OMAIN_CONTAINING_SIGNALING_PATHWAY							
GO_HINDLIMB_MORPHOGENESIS	3/129	37/17901	0.0024	0.2029	0.1873	FGF8/OSR2/PITX1	3
GO_POSITIVE_REGULATION_OF_PATTERN_RECOGNITION_RECEPTOR_SIGNALING_PATHWAY	3/129	37/17901	0.0024	0.2029	0.1873	HSPA1A/HSPA1B/ZCCHC3	3
GO_REGULATION_OF_NUCLEAR_DIVISION	6/129	188/17901	0.0024	0.2029	0.1873	FGF8/HSPA1A/HSPA1B/RIOK2/WNT4/ZW10	6
GO_ANDROGEN_BIOSYNTHETIC_PROCESS	2/129	11/17901	0.0027	0.2029	0.1873	HSD17B6/WNT4	2
GO_NEGATIVE_REGULATION_OF_INCLUSION_BODY_ASSEMBLY	2/129	11/17901	0.0027	0.2029	0.1873	HSPA1A/HSPA1B	2
GO_POSITIVE_REGULATION_OF_TUMOR_NECROSIS_FACTOR_MEDIATED_SIGNALING_PATHWAY	2/129	11/17901	0.0027	0.2029	0.1873	HSPA1A/HSPA1B	2
GO_REGULATION_OF_PROTEIN_EXPORT_FROM_NUCLEUS	3/129	41/17901	0.0032	0.2189	0.2020	DNAJC27/IFI27/RIOK2	3
GO_REGULATION_OF_MICROTUBULE_POLYMERIZATION_OR_DEPOLYMERIZATION	4/129	85/17901	0.0033	0.2189	0.2020	HSPA1A/HSPA1B/MAPRE1/SKA2	4
GO_PATTERN_RECOGNITION_RECEPTOR_SIGNALING_PATHWAY	6/129	201/17901	0.0034	0.2189	0.2020	HSPA1A/HSPA1B/HSPD1/S100A14/TLR6/ZCCHC3	6
GO_PITUITARY_GLAND_DEVELOPMENT	3/129	42/17901	0.0034	0.2189	0.2020	FGF8/PITX1/WNT4	3
GO_ADENOHYPOPHYSIS_DEVELOPMENT	2/129	13/17901	0.0038	0.2314	0.2135	FGF8/WNT4	2
GO_VESICLE_DOCKING_INVOLVED_IN_EXOCYTOSIS	3/129	44/17901	0.0039	0.2314	0.2135	CPLX2/EXOC6/VPS33A	3
GO_ESTABLISHMENT_OF_ORGANELLE_LOCALIZATION	9/129	432/17901	0.0041	0.2314	0.2135	BICD2/C17orf75/CPLX2/F8A3/KIF1C/MYO1F/RIOK2/SAPCD2/ZW10	9
GO_RESPONSE_TO_HYDROGEN_PEROXIDE	5/129	146/17901	0.0041	0.2314	0.2135	AQP1/HSPD1/KLF2/RHOB/RNF112	5
GO_NUCLEOTIDE_BINDING_OLIGOMERIZATION_DOMAIN_CONTAINING_2_SIGNALING_PATHWAY	2/129	14/17901	0.0044	0.2407	0.2222	HSPA1A/HSPA1B	2
GO_INTERACTION_WITH_SYMBIONT	4/129	93/17901	0.0046	0.2417	0.2231	AQP1/HSPD1/IFI27/PSMC3	4
GO_RESPONSE_TO_FIBROBLAST_GROWTH_FACTOR	5/129	153/17901	0.0050	0.2578	0.2379	EGR3/FGF8/HNRNPM/POLR2D/WNT4	5
GO_REGULATION_OF_PATTERN_RECOGNITION_RECEPTOR_SIGNALING_PATHWAY	4/129	99/17901	0.0057	0.2619	0.2417	HSPA1A/HSPA1B/TLR6/ZCCHC3	4
GO_NEGATIVE_REGULATION_OF_	2/129	16/17901	0.0058	0.2619	0.2417	ADAM15/NKX3-1	2

RECEPTOR_BINDING							
GO_REGULATION_OF_INCLUSION_BODY_ASSEMBLY	2/129	16/17901	0.0058	0.2619	0.2417	HSPA1A/HSPA1B	2
GO_REGULATION_OF_MICROTUBULE_POLYMERIZATION	3/129	51/17901	0.0059	0.2619	0.2417	HSPA1A/HSPA1B/MAPRE1	3
GO_CELLULAR_RESPONSE_TO_HYDROGEN_PEROXIDE	4/129	100/17901	0.0059	0.2619	0.2417	AQP1/KLF2/RHOB/RNF112	4
GO_DEVELOPMENT_OF_PRIMARY_SEXUAL_CHARACTERISTICS	6/129	229/17901	0.0063	0.2619	0.2417	ADAM15/FGF8/NKX3-1/PRDX4/VGF/WNT4	6
GO_NEGATIVE_REGULATION_OF_ALCOHOL_BIOSYNTHETIC_PROCESS	2/129	17/17901	0.0065	0.2619	0.2417	CYP27B1/ERLIN2	2
GO_REGULATION_OF_MITOTIC_SPINDLE_ASSEMBLY	2/129	17/17901	0.0065	0.2619	0.2417	HSPA1A/HSPA1B	2
GO_MESONEPHROS_DEVELOPMENT	4/129	103/17901	0.0066	0.2619	0.2417	FGF8/GDF11/OSR2/WNT4	4
GO_ENDOSOME_TO_LYSOSOME_TRANSPORT	3/129	53/17901	0.0066	0.2619	0.2417	RHOB/SNX16/VPS33A	3
GO_MALE_SEX_DIFFERENTIATION	5/129	164/17901	0.0067	0.2619	0.2417	ADAM15/FGF8/NKX3-1/PRDX4/WNT4	5
GO_RESPONSE_TO_REACTIVE_OXYGEN_SPECIES	6/129	235/17901	0.0072	0.2731	0.2520	AQP1/CYP2E1/HSPD1/KLF2/RHOB/RNF112	6
GO_CLATHRIN_COAT_ASSEMBLY	2/129	18/17901	0.0073	0.2731	0.2520	CALY/CLTB	2
GO_LYSOSOMAL_TRANSPORT	4/129	110/17901	0.0083	0.3021	0.2788	HSPA1A/RHOB/SNX16/VPS33A	4
